# On the Role of Anions in Solid Catalysts with Ionic Liquid Layer (SCILL) for the Selective Hydrogenation of Highly Concentrated Acetylene Streams

**DOI:** 10.1002/cssc.202401593

**Published:** 2024-10-21

**Authors:** Jonathan M. Mauß, Ferdi Schüth

**Affiliations:** ^1^ Department of Heterogeneous Catalysis Max-Planck-Institut für Kohlenforschung Kaiser-Wilhelm-Platz 1 45740 Mülheim an der Ruhr

**Keywords:** Methane pyrolysis, Selective hydrogenation, Acetylene, Ethylene, Ionic liquids

## Abstract

Electric plasma assisted pyrolysis of methane represents a highly promising greener alternative to produce ethylene from biogas and renewable energies compared to conventional steam cracking of naphtha. The mediocre performance of typical Pd−Ag catalysts for the downstream purification of the substantially higher concentrated acetylene impurities (≥15 vol.‐%) in those ethylene streams via selective hydrogenation is yet limiting economic interest. Following the concept of solid catalysts with ionic liquid layer (SCILL), we have modified an intrinsically non‐selective palladium catalyst with imidazolium based ionic liquids varying among 10 different anions and investigated them in this reaction. The best performing [C_4_C_1_IM][MeSO_4_]‐SCILL reaches an outstanding average ethylene selectivity over 20 h on‐stream of 82 % at full acetylene conversion without any sign of deactivation, clearly outperforming conventional Pd−Ag catalysts. By varying parameters like ionic liquid (IL) loading, temperature, feed gas composition, cations, and by using XPS for surface analysis we could gain a very comprehensive understanding of the underlying mechanisms that reduce the competing over‐hydrogenation and oligomerisation side‐reactions.

## Graphical abstract

Ionic liquids coated on the surface of solid Pd catalysts are highly potent promotors for ethylene selectivity in the selective hydrogenation of concentrated acetylene streams originating from the plasma activation of methane. Results of this study shed light on the underlying mechanism including reactant gas solubility, solvation shells and Pd site isolation phenomena being highly dependent on the chosen anion.

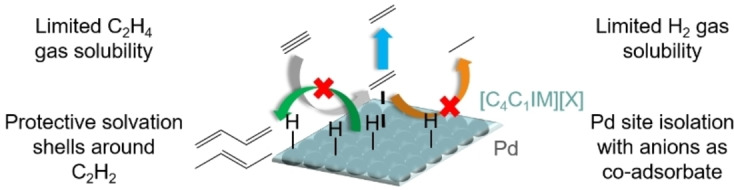

## Introduction

Ethylene is one of the most important platform molecules in the chemical industry, used for – amongst others – the production of polyethylene, 1,2‐dichloroethane, ethylene oxide, and ethylbenzene.[^1^, ^2^] In 2022, the global production capacity of ethylene exceeded 225 million metric tons.[^3^, ^4^] Today, ethylene is produced almost exclusively via the steam cracking of naphtha, resulting in the generation of up to 1.6 t CO_2_/t ethylene and additionally nitrogen oxides.^[1]^ Due to these emissions and the rising price of carbon dioxide emission certificates in Europe “greener” alternatives find increased interest.[^5^, ^6^] Such an alternative is ethylene production using an electric plasma‐assisted pyrolysis process of methane, known as the Hüls arc process. The methane and electricity required for the process can be obtained from sustainable sources like biogas and renewable energies, respectively. Ethylene production via this pathway could be used as a load leveler for the fluctuating performance of renewable energies in the future electricity grid.[^2^, ^7^, ^8^] The ethylene obtained via this process contains, however, significantly higher quantities of acetylene (≥15 vol.‐%) compared to ethylene coming from the steam cracking of naphtha (<2 vol.‐%).[^4^, ^7^] Since acetylene acts as a catalyst poison on the polymerization catalysts for ethylene, it must be removed almost completely (<5 ppm)^[9]^ from the ethylene stream.[^4^, ^9^, ^10^]

The economically most interesting purification method is the selective gas‐phase hydrogenation of acetylene to ethylene.[^4^, ^11^] Supported palladium catalysts show the best compromise of hydrogenation activity and selectivity to ethylene. Nevertheless, at higher acetylene conversions and poor temperature control, pristine palladium catalysts show low ethylene selectivity and stability due to over‐hydrogenation to ethane and the formation of oligomeric species.[^2^, ^4^] Very similar energy barriers for desorption of ethylene and its consecutive hydrogenation to ethane are suggested as the main reason for low ethylene selectivity of pure palladium catalysts.^[12]^ The most effective method for improving ethylene selectivity is the introduction of promoters consisting of an inert component or a second metal (e. g. Ag, Cu, Au). The selectivity improvement of these promoters is attributed to a spatial separation and size reduction of active palladium clusters (site‐isolation), whereby weaker ethylene adsorption modes are preferred. Furthermore, these promoters prevent the formation of very active but non‐selective palladium hydride phases.[^2^, ^13^, ^14^] At higher acetylene concentrations (>2 vol.‐%) than currently applied in industry such catalysts suffer from oligomerization side‐reactions. In addition, temperature control is difficult due to the high exothermicity of the reaction. We have previously demonstrated that conventional wet‐impregnated Pd−Ag catalysts reach an average ethylene selectivity at full acetylene conversion of up to 71 % (S C_2_H_6_ ~15 %, S C_>2_ ~14 %) and lifetimes up to 10 h at acetylene concentrations of 14 vol.‐%.^[15]^ However, this still appears to be insufficient for large scale application; in addition, oligomer formation (red and green oils) has to be suppressed to a larger extent.

Fine‐tuning the solubility of acetylene and ethylene in suitable ionic liquids (ILs), which have received high attention in recent years,^[16]^ appeared to be an option to improve catalyst performance. Palgunadi and coworkers demonstrated much higher gas solubility for acetylene than ethylene in some ILs. The acetylene/ethylene gas solubility ratio strongly correlates with the hydrogen bond acceptor ability of the anion while the choice of cation is of much less importance.[^17^, ^18^, ^19^, ^20^] In consecutive catalytic hydrogenation reactions these differences in the solubility of reactants, intermediates and products can be used to increase the selectivity to the semi‐hydrogenated intermediate.[^21^, ^22^, ^23^] Kernchen and coworkers deposited a thin layer of [C_4_C_1_IM][n‐C_8_H_17_OSO_3_] on a commercial porous nickel catalyst, which increased the yield in the consecutive liquid‐phase hydrogenation of clyclooctadiene to cyclooctene from 40 to 70 %.[^22^, ^24^] Applied to the selective gas‐phase hydrogenation of diluted acetylene streams (1 vol.‐%), Herrmann and coworkers were able to improve the ethylene selectivity of commercial Pd−Ag shell catalysts after depositing 5 w.–% [C_1_C_1_IM][MeHPO_3_] from 79 to 83 % by reducing over‐hydrogenation and oligomerization. However, the improvement in ethylene selectivity was accompanied by a significant drop in acetylene conversion (from 42 to 15 %), leading to ambiguous results.^[25]^ Several independent studies clearly confirmed the ethylene selectivity improvement effects reported by Herrmann and coworkers on similar Pd(Ag)‐based SILP and SCILL catalysts, even at constantly higher acetylene conversions.[^11^, ^26^, ^27^, ^28^] However, the range of ILs studied is too narrow for a conclusive attribution of the improved ethylene selectivity to solubility differences. The work of Xu and coworkers, for example, demonstrates that effects other than the gas solubility ratio of acetylene to ethylene may also have an influence on ethylene selectivity.^[29]^

Here we are aiming at elucidating the effect of different anions in imidazolium‐based ILs deposited on a solid porous palladium catalyst on ethylene selectivity in the selective gas‐phase hydrogenation of highly concentrated acetylene streams at industrially relevant high pressure. We show that surface modification of palladium catalysts with selected ILs improves ethylene selectivity and catalyst stability under those harsh reaction conditions more effectively than silver.

## Results and Discussion

### Catalyst Characterization

**Figure 1:**
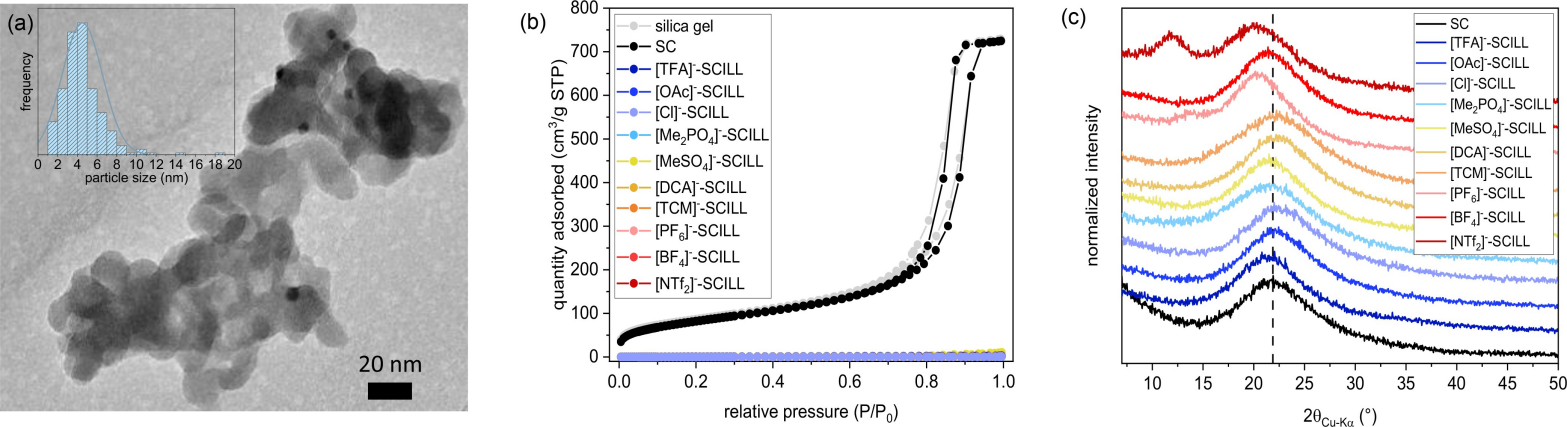
(a) TEM image of SC with particle size distribution (N=277). (b) N_2_ physisorption isotherms of SC and corresponding SCILL catalysts with various anions based on [C_4_C_1_IM]^+^ containing ionic liquids. (c) XRD pattern of SC and corresponding SCILL catalysts with various anions at low angles. Vertical line is at the position of the broad reflection of the silica gel support.

The metal loading of the Pd/silica gel catalyst (SC) after reduction and annealing determined via ICP‐OES (0.09 w.–%) corresponds within the expected precision to the intended 0.1 w.–%. Due to the low metal loading no reflections of Pd were observed in XRD analysis (Figure S1). TEM images (Figure 1a) reveal a quite narrow particle size distribution with most particles being in the range of 3–7 nm. In nitrogen physisorption analysis (Figure 1b), SC displays, in comparison to the pristine silica gel support, an almost identical pore size of about 15 nm (BJH desorption, isotherm type IV, Figure S2) with a pore volume of 1.12 cm^3^/g and a BET surface area of 302 m^2^/g. After deposition of the imidazolium based ILs in the pore system of SC, the adsorbed amount of nitrogen is drastically reduced and the isotherm hysteresis has disappeared. Overall, the obtained SCILLs exhibit typical features for non‐porous materials with very low BET surface areas <3 m^2^/g and pore volumes <0.02 cm^3^/g. Further details are summarized in the supporting information (Table S1). To further prove the complete pore filling of the silica gel with ILs, the weight loading of ILs in the SCILLs was controlled via TGA and SEM‐EDX bulk measurements (Tables S2–S3). Nominal deviations in weight loss measured via TGA from calculated values were less than 5 % and are most likely caused by water and other organic impurities (cf. purity of ILs >95 %). Moreover, in some cases, there were indications of incomplete combustion to residual coke, as the samples turned from greyish to black after measurement. This may be due to the encapsulation of the IL inside the pores as well as to the formation of non‐volatile inorganic residues (e. g. phosphates, halogenides). All expected elements in the so prepared SCILLs could be found via SEM‐EDX, and the concentrations matched approximately the expected values. The formation of a continuous liquid phase of the ILs in the pore system of the support for some of the SCILLs is also indicated by additional very broad reflections at low angles in the XRD patterns (Figures 1c and S3). For example, in case of the [C_4_C_1_IM][NTf_2_]‐SCILL these additional reflections appear at 12.1 and 19.3°. The short‐range ordering of cations and anions of this ionic liquid in thin films of a few nanometers on silica surfaces has already been reported by Tomita and coworkers, based on synchrotron X‐ray diffraction measurements.^[30]^

### Catalytic Testing

**Figure 2:**
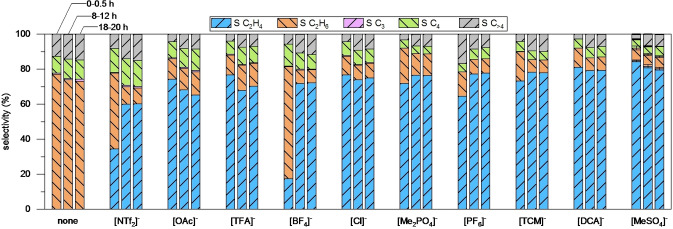
Average ethylene, ethane, C_3_, C_4_ and C_>4_ selectivity of Pd/silica gel solid catalyst (SC/none) and corresponding [C_4_C_1_IM]^+^ based SCILL catalysts with various anions at different time‐on‐stream intervals (0–0.5 h, 8–12 h and 18–20 h) in the selective hydrogenation of concentrated acetylene streams (C_2_H_2_/C_2_H_4_/H_2_ 1 : 1 : 5, 10 bar, 120 °C, X_C2H2_=100 %, CB≥0.93, WHSV 42 000 cm^3^ h^−1^ g^−1^_cat_). Complete hydrogenation of co‐fed ethylene in the case of SC were corrected in the selectivity to ethylene and ethane for better comparison (see text).

The 0.1 w.–% Pd/silica gel catalyst (SC) and the corresponding SCILLs with different anions were tested in the selective gas‐phase hydrogenation of concentrated acetylene streams (C_2_H_2_/C_2_H_4_/H_2_ 1 : 1 : 5) at 120 °C and 10 bar pressure. Figure 2 depicts the average ethylene and ethane selectivity as well as the selectivity of C_3_ (propane, propene), C_4_ (butane, 1‐butene, 2‐cis‐butene, 2‐trans‐butene) and C_>4_ compounds at full acetylene conversion at three different time‐on‐stream intervals (0–0.5 h, 8–12 h and 18–20 h). More detailed catalytic performance data can be found in Figures S4–S14 in the supporting information. For all time‐on‐stream intervals, the SC exhibits no selectivity to ethylene under the chosen reaction conditions. Even the co‐fed ethylene is completely hydrogenated to ethane, leading to calculated ethylene and ethane selectivities of −100 and +177 %, respectively (Figure S4). As no side‐products other than ethane are observable in ethylene hydrogenation with SC under analogous reaction conditions without acetylene (Figures 4a and S34), the ethylene and ethane selectivity in Figure 2 were corrected to 0 % and +77 %, respectively, for easier comparison. This non‐selective behavior of SC can be explained by the high exothermicity of the sequential hydrogenation reaction, causing pronounced hotspot formation in the catalyst bed at such high acetylene concentrations. The axial temperature profile measured in the catalyst bed during reaction at 120 °C oven temperature reveals temperatures around 184, 163 and 150 °C in the top, middle and bottom bed position (Figure S4). Lowering of the oven temperature to 60 °C does not lead to an improvement in ethylene selectivity in case of SC. Higher temperatures (160 °C) decrease ethane formation of SC but increase selectivity to oligomerization products (C_>2_) rather than to ethylene (Figures S15–S16). These observations underline the difficulties of catalysts to become selective at higher acetylene concentrations (>2 vol.‐%). If used in the hydrogenation of diluted acetylene streams (1 vol.‐%) at 60 °C, on the other hand, SC displays an ethylene selectivity of up to 24 % without formation of extreme hotspots (<20 °C, Figures S16–S17) comparable to similarly prepared catalysts reported in literature.[^11^, ^31^, ^32^] In order to study the effect of anions in the SCILLs for the selective hydrogenation of concentrated acetylene streams, anions X in [C_4_C_1_IM][X] type ILs were selected, which had already been object of other studies (e. g. [NTf_2_]^−^, [BF_4_]^−^, [Cl]^−^, [PF_6_]^−^, [DCA]^−^),[^11^, ^25^, ^26^, ^27^, ^28^, ^29^] as well as promising additional examples, which have not yet been investigated in literature (e. g. [OAc]^−^, [TFA]^−^, [Me_2_PO_4_]^−^, [TCM]^−^, [MeSO_4_]^−^). After modifying SC with ILs to SCILLs, the ethylene selectivity is significantly improved for all selected anions X at identical reaction conditions and complete acetylene conversion, but to very different extents. While the [BF_4_]^−^‐SCILL exhibits, for example, an ethylene selectivity of 17 % during the first half hour on‐stream, the [MeSO_4_]^−^‐SCILL converts acetylene with a selectivity of 85 % to ethylene during the same period. Averaged over 20 hours on‐stream the ethylene selectivity improves in the order SC<[NTf_2_]^−^‐<[OAc]^−^‐<[TFA]^−^‐<[BF_4_]^−^‐<[Cl]^−^‐<[Me_2_PO_4_]^−^‐<[PF_6_]^−^‐<[TCM]^−^‐<[DCA]^−^‐<[MeSO_4_]^−^‐SCILL. This improvement in ethylene selectivity of SCILLs is mainly caused by a remarkable reduction of the sequential hydrogenation side‐reaction to ethane from 177 % (SC) down to 7 % ([MeSO_4_]^−^‐SCILL). Except for [NTf_2_]^−^‐SCILL, however, also the selectivity to oligomerisation products (sum C_>2_) decreases from 23 % (SC) to 8 % ([Me_2_PO_4_]^−^‐, [DCA]^−^‐ and [MeSO_4_]^−^‐SCILL). Interestingly, the inhibition of one of the two side‐reactions, over‐hydrogenation to ethane and oligomerisation to C_>2_, is very different from one SCILL to the other. [Me_2_PO_4_]^−^‐SCILL, for example, displays an average ethylene selectivity of 72 %, an ethane selectivity of 20 % and a C_>2_ selectivity of 8 % during the first half hour on‐stream. [OAc]^−^‐SCILL, on the other hand, exhibits a quite similar ethylene selectivity of 74 % in the same interval but with a much lower ethane selectivity of 12 % and a much higher C_>2_ selectivity of 14 %. This observation already suggests that there might be more than one specific parameter of ionic liquids that affects the ethylene selectivity performance of SCILLs. Overall, the ethylene selectivity performance of most SCILLs is rather constant from the beginning on during 20 h on‐stream. [NTf_2_]^−^‐, [BF_4_]^−^‐ and [PF_6_]^−^‐SCILL, however, improve significantly in ethylene selectivity within the first hours on‐stream until a plateau value is reached. Since those SCILLs also have a comparably high oligomerization selectivity, this phenomenon can most likely be explained by the deactivation of very active but non‐selective Pd sites by high‐boiling oligomeric species, suppressing further hydrogenation of adsorbed ethylene.[^2^, ^33^] To a smaller extent, a similar effect can also be observed for SC and others SCILLs as the selectivity to oligomers (C_>2_) is slightly increasing with time‐on‐stream, while selectivity to ethane is simultaneously decreasing. Besides that, ethylene selectivity for [OAc]^−^‐, [TFA]^−^‐, [Cl]^−^‐, [DCA]^−^‐ and [MeSO_4_]^−^‐SCILL is slightly decreasing with time‐on‐stream.

**Figure 3:**
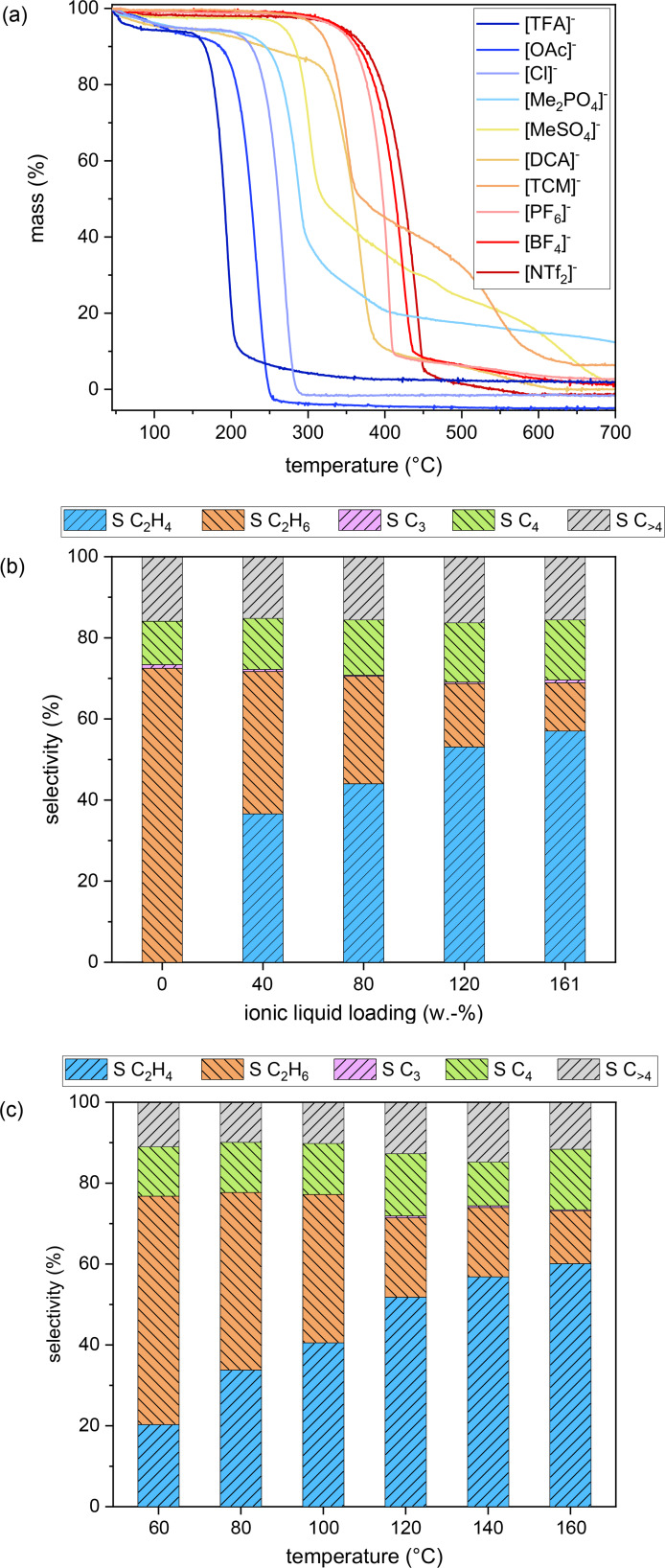
(a) TGA decomposition curves of [C_4_C_1_IM]^+^ based ionic liquids with different anions at 5 °C/min heating rate and 40 mL/min air, average ethylene, ethane, C_3_, C_4_ and C_>4_ selectivity of [NTf_2_]^−^‐SCILL (C_2_H_2_/C_2_H_4_/H_2_ 1 : 1 : 5, 10 bar, 120 °C, X_C2H2_=100 %, CB≥0.93, WHSV 42 000 cm^3^ h^−1^ g^−1^_cat_) under variation of (b) ionic liquid loading (0–20 h on‐stream) and (c) temperature (0–5 h on‐stream).

As some ionic liquids are known to have a limited thermal stability,[^34^, ^35^, ^36^] we conducted TGA measurements of the unsupported ILs (and SCILLS) in order to assess, if the selectivity loss correlates with the decomposition of the IL (Figures 3a and S18). Temperatures for 50 % decomposition of the IL (T_dec,50 %_) vary across a wide range from 191 °C to 426 °C in the order [TFA]^−^<[OAc]^−^<[Cl]^−^<[Me_2_PO_4_]^−^<[MeSO_4_]^−^<[DCA]^−^<[TCM]^−^<[PF_6_]^−^<[BF_4_]^−^<[NTf_2_]^−^. As decomposition kinetics of ILs are known to be sluggish,[^35^, ^36^] we also conducted long‐term stability tests at 150, 200 and 250 °C (Figures S19–S21). All data is summarized in Table S4 in the supporting information. Despite the comparably high decomposition temperature (T_dec,50 %_ ~ 191 °C) in initial TGA measurements, the isothermal longterm experiments reveal already a pronounced mass loss (−84.4 %) for [C_4_C_1_IM][TFA] at 150 °C after 5 hours. Thermally more stable ionic liquids with anions [MeSO_4_]^−^, [DCA]^−^, [PF_6_]^−^, [NTf_2_]^−^, on the other hand, exhibit high stability at 150 and 200 °C. In the case of [MeSO_4_]^−^ and [NTf_2_]^−^ this thermal stability extends even up to 250 °C. In the case of [TFA]^−^‐ and [OAc]^−^‐SCILL it seems thus likely that ethylene selectivity is decreasing over time‐on‐stream due to the decomposition of the ionic liquid. The other SCILL catalysts, however, are very likely to be stable under reaction conditions. An unexplored possibility for decomposition of the ILs is a Pd catalyzed hydrogenolysis side reaction of the ionic liquids in SCILLs under reaction conditions, which cannot be easily studied in the TGA due to coking under such conditions. However, no traces of such potential hydrogenolysis products were detected with the sensitive GC systems used.

In a previous study of our group Pd_x_Ag_y_ alloyed catalysts of different compositions were optimized for the selective hydrogenation of such concentrated acetylene streams.^[15]^ For comparison to the SCILLs in this study, we have re‐synthesized the best performing composition Pd_1_Ag_9_ on silica gel. This catalyst exhibits, similar as in the former study, an average ethylene, ethane and C_>2_ selectivity of 68 %, 11 % and 21 % over 13 hours on‐stream (Figure S22). Except [NTf_2_]^−^‐SCILL, all SCILLs show (much) higher average ethylene selectivities of up to 82 % ([MeSO_4_]^−^‐SCILL), demonstrating the outstanding performance of those catalysts under these harsh reaction conditions. Moreover, the Pd_1_Ag_9_/silica gel solid catalyst, while having approximately the same Pd loading (~0.1 w.–%) as SC and SCILLs, deactivates after 13 hours on‐stream accompanied by a pronounced temperature shift in the catalyst bed indicating an axial migration of the hotspot with deactivation (cf. Figure S23). In contrast, SC and SCILLs are active for >20 hours on‐stream, and no shift in the axial temperature profile of the catalyst bed is observed. It might be possible that the extended ionic liquid phase in the pore system of the SCILLs removes high‐boiling oligomers from the catalyst surface via dissolution and thereby increases the lifetime on‐stream.

### Origin of Selectivity Enhancement

In order to elucidate the reasons for the significant ethylene selectivity improvement from SC to SCILLs and its high dependency on the chosen anion, the underlying phenomena were further investigated. For diluted acetylene streams, improvements in ethylene selectivity by surface modifications with ILs are so far mostly explained by a high acetylene to ethylene gas solubility ratio of the chosen IL.[^11^, ^25^, ^26^, ^27^, ^28^, ^29^] In our case, the correlation between the observed ethylene selectivity of SCILLs and the reported acetylene/ethylene gas solubility ratios α for the pure ionic liquids is very poor (Table S5).[^11^, ^19^, ^20^, ^26^] While the α value increases from 4 ([C_4_C_1_IM][NTf_2_]) to 28 ([C_4_C_1_IM][OAc]) in the order [NTf_2_]^−^<[DCA]^−^<[TFA]^−^<[PF_6_]^−^~ [BF_4_]^−^<[Cl]^−^<[MeSO_4_]^−^<[Me_2_PO_4_]^−^<[OAc]^−^, the observed average ethylene selectivity over 20 h on‐stream improves in the order [NTf_2_]^−^<[OAc]^−^~ [TFA]^−^<[BF_4_]^−^<[Cl]^−^<[Me_2_PO_4_]^−^<[PF_6_]^−^<[DCA]^−^<[MeSO_4_]^−^. Consequently, the performance of an ionic liquid in a SCILL system does not seem to be simply predictable by the gas solubility ratio, but results most likely from a complex interplay of different properties. In the following, several control experiments with the thermally most stable [NTf_2_]^−^‐SCILL are reported in order to more comprehensively assess the impact of different parameters on the performance of the SCILLs in this reaction.

### Ionic Liquid Loading

At first the loading of IL in the [NTf_2_]^−^‐SCILL was varied from 0 (SC) to 161 w.–%, with 161 w.–% loading corresponding to complete filling of the pore system, taking into account the bulk density of the IL. Corresponding characterization data can be found in the supporting information (Figures S24–S25, Tables S6–S8). Under the same reaction conditions as before the average ethylene selectivity over 20 hours on‐stream significantly improves with the weight loading of IL from 37 % (40 w.–%) to 57 % (161 w.–%) (Figures 3b, S4–S5, S26–S28). Diffusion pathways of reactant gases (acetylene, ethylene and hydrogen) through the IL layer increase with the pore filling of the support. A simultaneous ethylene selectivity enhancement indicates that gas solubility might indeed play an important role. Limited availability of hydrogen as well as slow mass transfer kinetics on the catalyst surface could explain the slightly increasing selectivity to oligomeric species with increasing IL loading. Reduced full hydrogenation to ethane can be rationalized by a decreased availability of ethylene or hydrogen.

### Temperature

In order to decouple effects of limited mass transfer and reactant gas solubility, the reactor temperature for [NTf_2_]^−^‐SCILL (161 w.–% IL) was varied from 60 to 160 °C (Figures 3c, S5, and S29–S33). Increasing the reaction temperature increases the ethylene selectivity from 20 % (60 °C) to 60 % (160 °C) by reducing full hydrogenation to ethane but slightly increasing formation of oligomeric species in the first five hours on‐stream. Diffusion processes in the IL governed by the motion of the ions accelerate with temperature. Consequently, the replenishment of hydrogen on the surface should proceed faster with increasing temperature. Reduced full hydrogenation and increased oligomerization at higher temperatures, however, indicate inversely a lower availability of activated hydrogen species at active Pd sites. It is thus rather unlikely that temperature‐dependent variations in mass transfer kinetics play a role. Gas solubility, on the other hand, is known to generally decrease with increasing temperature in (ionic) liquids, which would fit to the observed selectivity trends. With regard to the influence of temperature on hydrogen solubility in ILs, fundamentally different observations have so far been reported in the literature.[^37^, ^38^, ^39^, ^40^, ^41^] Consequently, it cannot clearly be decided, if the ethylene selectivity improvement is related to a lowered ethylene or hydrogen availability on catalytically active Pd sites.

### Addition of Acetylene and Ethylene

**Figure 4:**
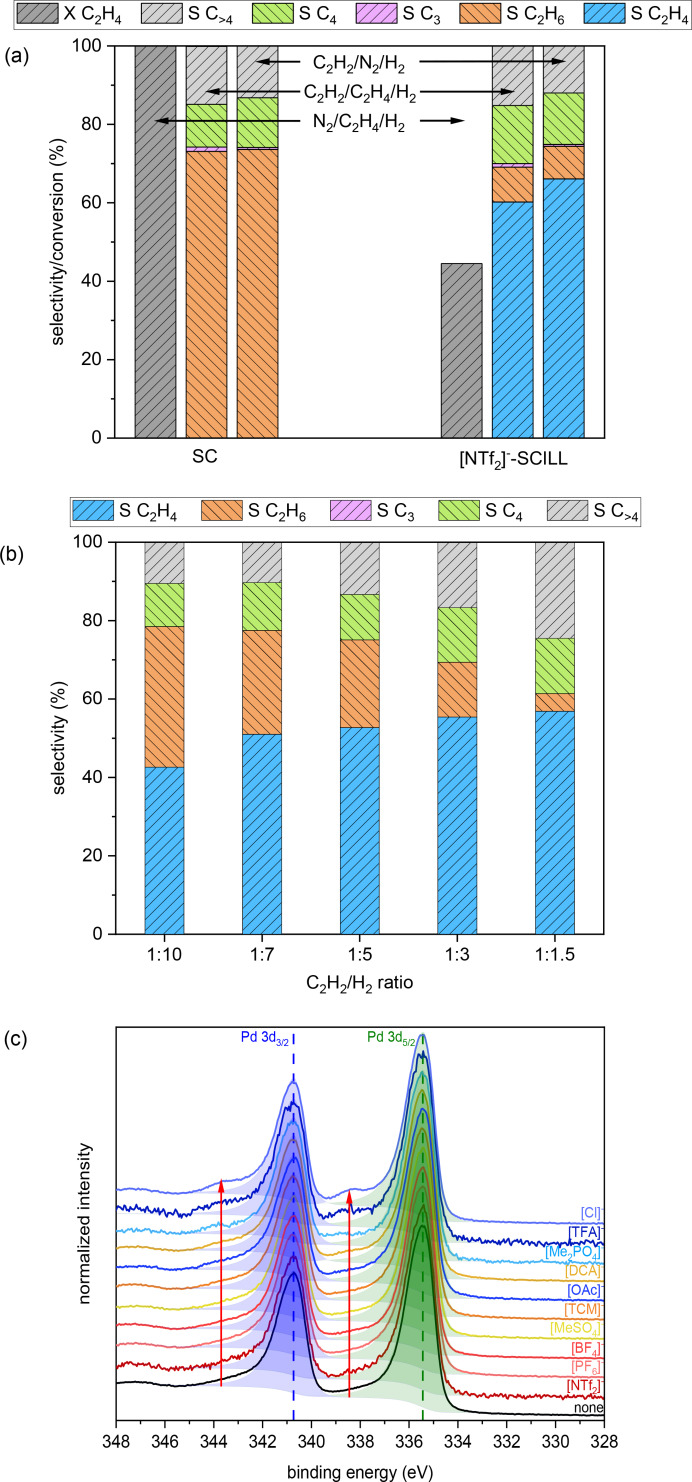
Average ethylene, ethane, C_3_, C_4_ and C_>4_ selectivity of [NTf_2_]^−^‐SCILL (10 bar, 120 °C, X_C2H2_=100 %) under variation of (a) acetylene/ethylene feed gas composition (15–20 h on‐stream, C_2_H_2_ or N_2_/C_2_H_4_ or N_2_/H_2_ 1 : 1 : 5, CB≥0.89, WHSV 42 000 cm^3^ h^−1^ g^−1^_cat_), (b) hydrogen feed gas composition (15–20 h on‐stream, C_2_H_2_/C_2_H_4_/H_2_ and N_2_ 1 : 1 : 10, CB≥0.87, WHSV 36 000 cm^3^ h^−1^ g^−1^_cat_), (c) XPS spectra of Pd 3d region of Pd model catalysts surface coated with [C_4_C_1_IM]^+^ containing ionic liquids. Red arrow indicate increasing intensity of an oxidized Pd species at higher binding energies depending on the anion.

In order to distinguish the effect of ethylene and hydrogen availability, either ethylene or acetylene was replaced in the feed gas by nitrogen, maintaining the same overall feed gas flow rate and amount of hydrogen. The results were compared with the results of reactions during co‐feeding ethylene and acetylene in the case of SC and [NTf_2_]^−^‐SCILL (Figures 4a, S4–S5, and S34–S37). In case of SC, independent of the feed gas composition, no ethylene can be found in the product stream. The selectivity pattern when acetylene was present in the feed is almost identical. Only selectivity to oligomeric species is slightly increased when acetylene and ethylene are co‐fed. [NTf_2_]^−^‐SCILL, on the other hand, exhibits a significantly different catalytic performance depending on the feed gas composition. In the acetylene‐free feed, [NTf_2_]^−^‐SCILL is significantly less prone to hydrogenate ethylene (X C_2_H_4_ ~ 45 %) than SC (X C_2_H_4_=100 %). This decreased hydrogenation activity of ethylene under reaction conditions might be rationalized by a decreased availability of ethylene and/or hydrogen. Henry volatility constants, scaling inversely with the solubility of a gas in a liquid, are, however, two orders of magnitude higher for hydrogen (450 MPa) than for ethylene (8.29 MPa) in [C_4_C_1_IM][NTf_2_] (Table S5).[^19^, ^42^] Since hydrogen and ethylene are required in an equimolar ratio for the hydrogenation reaction, it is thus much more likely that a lack of hydrogen in the IL is causing a decreased hydrogenation activity of ethylene. Having the same amount of hydrogen but no ethylene in the feed does indeed not change the ethane selectivity (8–9 %) of [NTf_2_]^−^‐SCILL when compared to the co‐fed reaction. This demonstrates that a lower availability of ethylene does not affect the full hydrogenation side‐reaction. Nevertheless, [NTf_2_]^−^‐SCILL displays overall a higher ethylene selectivity in the ethylene free feed (66 vs. 60 %) compared to the co‐fed reaction. This ethylene selectivity improvement is caused by an equally reduced oligomerization selectivity (26 vs. 31 %). The cooperative role of ethylene in oligomerisation processes of acetylene during the hydrogenation reaction on Pd catalysts has already been reported in literature.^[33]^ In conclusion, it seems that the availability of hydrogen determines the full hydrogenation side‐reaction to ethane while the presence of ethylene impacts the oligomerization side‐reaction of acetylene. Using hydrogen solubility as a parameter for ethane selectivity of the previously tested SCILLs, it is indeed possible to find a correlation. While the average ethane selectivity over 20 h on‐stream decreases in the order [TFA]^−^‐>[BF_4_]^−^‐>[PF_6_]^−^‐SCILL also the Henry volatility constants of hydrogen of the corresponding ILs increase stepwise from 490 to 660 MPa (Table S5).^[42]^ At the same time, these ILs have approximately the same acetylene to ethylene gas solubility ratio α (10–11, Table S5)[^19^, ^26^] and very similar and much lower Henry volatility constants for ethylene (13.6–25.3 MPa, Table S5)[^18^, ^19^, ^26^]. A similar correlation between oligomerization selectivtiy and ethylene solubility in the IL is not obvious. Since a lack of hydrogen might also promote the oligomerization side‐reaction, it is possible that both effects interfere at this point. Taking into account also the experiments with varying ionic liquid loading and temperature, it is thus much more likely that a decreased availability of hydrogen rather than of ethylene is causing improved ethylene selectivity via reduced full hydrogenation to ethane, but slightly increased oligomerization tendency.

### Addition of Hydrogen

To further investigate the impact of hydrogen gas solubility on both side‐reactions, for [NTf_2_]^−^‐SCILL hydrogen was stepwise replaced by nitrogen, keeping the same amount of ethylene, to achieve decreasing C_2_H_2_/H_2_ ratios from 1 : 10–1 : 1.5 (Figures 4b and S38–S42). As expected ethylene selectivity increases with reduced availability of hydrogen from 43 to 57 % via reduced full hydrogenation to ethane from 36 to 5 % while the formation of oligomeric species is strongly increased from 11 to 25 %. Similar effects can also be observed by reducing the hydrogen gas solubility via shortening the alkyl chain length on the cation in the [Me_2_PO_4_]^−^‐SCILL (see details in supporting information, Tables S9–S13, Figures S10 and S43–S49). These observations underline the importance of hydrogen availability not only for the full hydrogenation side‐reaction to ethane, but also for the oligomerization side‐reaction.

### Viscosity, Hydrogen Bond Basicity and Anion Volume

Even with the knowledge on the influence of hydrogen gas solubility on both side‐reactions, the performance of some previously tested SCILLs can still not be fully rationalized. [Me_2_PO_4_]^−^‐, [PF_6_]^−^‐ and [MeSO_4_]^−^‐SCILL, for example, display high ethylene selectivity (77–82 %) but not only due to a very low ethane (7–13 %) but also low oligomerization selectivity (11–14 %). Variation of ethylene gas solubility in those ionic liquids compared to [C_4_C_1_IM][NTf_2_] is too low to explain the significantly reduced formation of oligomeric species with regard to [NTf_2_]^−^‐SCILL (30 %). Comparing different parameters, correlations between a reduced oligomerisation selectivity and high viscosity,[^43^, ^44^, ^45^] anion hydrogen bond basicity β^[46]^ and volume of the anion[^47^, ^54^] in the IL (Table S5) was discovered. The anion hydrogen bond basicity β is used as a scale for the strength of interaction between (basic) anions and slightly acidic hydrogen atoms, as for example in the acetylene molecule, and are deduced from observed solvochromatic shifts in UV‐Vis spectroscopy of a specific set of dyes dissolved in the IL.[^18^, ^46^, ^48^, ^49^] The observed correlation between those parameters suggests that solvation shells of anions (and cations) around the solubilized acetylene molecules in the IL might keep activated acetylenic species on catalytically active Pd sites away from each other. Higher viscosity, stronger anion‐acetylene interactions and more bulky anions increase this effect by the solvation shells, which might inhibit oligomerisation reactions to proceed on a Pd surface depleted of activated hydrogen. Low hydrogen solubility coupled with strong solvation shells around the acetylene molecule might explain simultaneous low ethane and oligomerization selectivity of the previously mentioned SCILLs.

### Electronic Effects

Solvation shell effects help to understand the low oligomerisation selectivity of [Cl]^−^‐, [Me_2_PO_4_]^−^‐, [PF_6_]^−^‐ and [MeSO_4_]^−^‐SCILLs, but some discrepancies remain for [TCM]^−^‐ and [DCA]^−^‐SCILLs. SCILLs based on both ionic liquids with cyanide moieties display simultaneously low oligomerisation selectivity and fairly low room‐temperature viscosities (<33 mPa s),^[43]^ lower hydrogen bond basicity values β (<0.64),^[46]^ and contain in case of [C_4_C_1_IM][DCA] a rather small anion (0.089 nm^3^).^[47]^ As many of the studied anions are known to be strong coordinators to metal centers, electronic effects might influence the selectivity in those SCILLs. In order to study electronic effects of the investigated anions on a Pd surface, model systems were synthesized by dip‐coating a Pd foil into ionic liquid/acetone solutions and analyzing the resulting thin films via XPS (Figures 4c and S46). Most of the ionic liquid‐Pd model systems have a very similar Pd 3d region as the pristine Pd foil, assigned to metallic palladium. In the case of the [OAc]^−^‐, [DCA]^−^‐ [Me_2_PO_4_]^−^‐, [TFA]^−^‐ and [Cl]^−^‐Pd model system, however, increasing contributions of additional, oxidized Pd species at higher binding energies (Δ>2 eV) can be clearly observed. Such additional oxidized Pd species, due to the interaction of Pd with ionic liquids like [C_4_C_1_IM][NTf_2_]/[DCA], have already been observed via XPS by Arras and coworkers for surface modified Pd/silica gel model systems.^[50]^ Strong coordination of those anions to the Pd surface might facilitate surface oxidation with atmospheric oxygen via stabilization of the resulting oxidized Pd^x+^ species. This phenomenon suggests that some anions coordinate more strongly to the Pd surface than others, thereby causing a Pd site‐isolation as co‐adsorbates in competition to ethylene and acetylene molecules, similar to the geometric effect in bimetallic Pd−Ag catalysts. Such electronic or active site blocking effects of specific anions might therefore be another important parameter to understand reduced overhydrogenation as well as oligomerisation processes of SCILLs.

## Conclusions

In this study, we demonstrate that SCILLs, based on modification of solid Pd catalysts with well‐chosen ionic liquids, can impressively improve the ethylene selectivity in the selective hydrogenation of concentrated ethylene/acetylene streams. Investigating and comparing a wide range of different anions for this reaction, it was revealed that the choice of anion in the IL has a strong impact on the selectivity performance of SCILLs. The best SCILL ([C_4_C_1_IM][MeSO_4_]‐SCILL) reaches an average ethylene selectivity of 82 % at full acetylene conversion over 20 hours on‐stream without showing any sign of deactivation. This system clearly outperforms conventional Pd−Ag catalysts with regard to ethylene selectivity and stability on‐stream under those harsh reaction conditions. By varying different parameters like IL loading, temperature, feed gas composition (with regard to ethylene, acetylene and hydrogen) and cations as well as examining electronic interactions with a Pd surface via XPS, a profound understanding was obtained on how ILs might improve the selectivity performance of the catalyst. The results clearly indicate that the gas solubility ratio of acetylene to ethylene in the IL, in contrast to statements in literature, only marginally affects the selectivity performance of SCILLs. Low hydrogen solubility coupled with strong and bulky protective solvation shells around the acetylene molecules in the IL are more likely responsible for reduced full hydrogenation and oligomerisation side‐reactions. Surface analysis via XPS additionally suggests that also co‐adsorption effects of anions on the Pd surface during catalysis in competition to acetylene, ethylene and hydrogen molecules might contribute to the selectivity enhancement of SCILLs. This effect could be similar to the geometric site‐isolation of Pd sites in bimetallic Pd−Ag catalysts. The results of this study demonstrate the high potential of ionic liquids to guide the reactivity of non‐selective solid catalysts into the wanted direction even under very unfavorable reaction conditions. Understanding the underlying phenomena of ionic liquids in SCILLs might pave the way to the design of ionic liquids with properties adapted to more complicated, molecule‐specific selective hydrogenation reactions.

## Supporting Information Summary

The authors have cited additional references within the Supporting Information (Ref. [11, 15, 18–20, 26, 37, 41–47, 51–54]).

## Conflict of Interests

The authors declare no conflict of interest.

1

## Supporting information

As a service to our authors and readers, this journal provides supporting information supplied by the authors. Such materials are peer reviewed and may be re‐organized for online delivery, but are not copy‐edited or typeset. Technical support issues arising from supporting information (other than missing files) should be addressed to the authors.

Supporting Information

## Data Availability

The data that support the findings of this study are available in the supplementary material of this article.
