# Supplementary material for: On the Role of Anions in Solid Catalysts with Ionic Liquid Layer (SCILL) for the Selective Hydrogenation of Highly Concentrated Acetylene Streams
Source: ChemSusChem. 2024 Oct 21;18(2):e202401593. doi: 10.1002/cssc.202401593 (PMC11739838; doi:10.1002/cssc.202401593)
Supplement: Supplementary file 1 — Supporting Information [file CSSC-18-e202401593-s001.pdf]

ChemSusChem

Supporting Information

On the Role of Anions in Solid Catalysts with Ionic Liquid Layer (SCILL) for the Selective Hydrogenation of Highly Concentrated Acetylene Streams

Jonathan M. Mauß and Ferdi Schüth*

Supporting Information

On the Role of Anions in Solid Catalysts with Ionic Liquid Layer (SCILL) for the Selective Hydrogenation of Highly Concentrated Acetylene Streams

Jonathan M. Mauß, Ferdi Schüth*

Max-Planck-Institut für Kohlenforschung, Department of Heterogeneous Catalysis, Kaiser-Wilhelm-Platz 1, 45470 Mülheim an der Ruhr, Germany

Experimental

Materials

Silica gel (Supleco, high-purity grade, Davisil Grade 62 or 643, pore size 150 Å), palladium (II) nitrate dihydrate (~40% Pd basis) and silver nitrate (> 99.9999% trace metals basis) were purchased from Sigma Aldrich and used as received. Quartz wool (Roth, chemically pure), silicon carbide (Alfa Aesar, 46 grit), nitric acid (J.T.Baker, conc., 65%, < 5 ppb Hg) and acetone (Sigma-Aldrich, > 99.9% HPLC purity grade) were used as obtained. 1-Butyl-3-methylimidazolium bis(trifluoromethylsulfonyl)imide ([C_4C_1IM][NTf_2], Sigma Aldrich, ≥ 98%), 1-Butyl-3-methylimidazolium acetate ([C_4C_1IM][OAc], TCI, > 95%), 1-Butyl-3-methylimidazolium trifluoroacetate ([C_4C_1IM][TFA], BLDpharm, > 97%), 1-Butyl-3-methylimidazolium tetrafluoroborate ([C_4C_1IM][BF_4], TCI, > 98%), 1-Butyl-3-methylimidazolium chloride ([C_4C_1IM][Cl], Iolitec, 99%), 1,3-Dimethylimidazolium dimethylphosphate ([C_1C_1IM][Me_2PO_4], Thermo Scientific, 98%), 1-Ethyl-3-methylimidazolium dimethylphosphate ([C_2C_1IM][Me_2PO_4], Sigma Aldrich, ≥ 98% HPLC),1-Butyl-3-methylimidazolium dimethylphosphate ([C_4C_1IM][Me_2PO_4], abcr, 98%), 1-Butyl-3-methylimidazolium hexafluorophosphate ([C_4C_1IM][PF_6], BLDpharm, 99.97%), 1-Butyl-3-methylimidazolium tricyanomethanide ([C_4C_1IM][TCM], TCI, > 98%), 1-Butyl-3-methylimidazolium dicyanamide ([C_4C_1IM][DCA], BLDpharm, 98%), 1-Butyl-3-methylimidazolium methylsulfate ([C_4C_1IM][$MeSO_4$], Sigma Aldrich, ≥ 97.0% HPLC) were used without further purification. Palladium foil (0.1 mm, > 99.99%) was obtained from GoodFellow GmbH and cleaned multiple times from organic impurities ultrasonically with acetone before usage.

Catalyst synthesis

Palladium nitrate dihydrate (and silver nitrate in case of Pd_1Ag_9) was dissolved in diluted nitric acid (1 mM, pH 3) to achieve a metal loading of 0.1 w.-% Pd (1 w.-% Pd_1Ag_9 molar ratio) on the silica gel support in a volume consistent with the pore volume of silica gel support (1.12 cm^3/g) under stirring and heating to 60 °C. The obtained solution was impregnated following the incipient wetness technique onto the previously dried silica gel support (150 °C, > 12 h). Afterwards, the impregnated material was dried at 75 °C (> 12 h), calcined at 120 °C (2 h, 2 °C/min) followed by reduction under pure hydrogen flow (100 mL/min) at 150 °C (3 h, 2 °C/min) and annealing under pure Ar flow (100 mL/min) at 600 °C (10 h for Pd, 20 h for Pd_1Ag_9, 10 °C/min). For the preparation of SCILLs (solid catalysts with ionic liquid layer), the ionic liquids were either taken purely or diluted with acetone to achieve different ionic liquid loadings using an impregnation volume adapted to the pore volume of the material (1.12 cm^3/g), incipient-wetness impregnated onto the previously dried material (> 12 h, 150 °C) and further dried afterwards at 80 °C (4 h). Impregnation with non-room temperature ionic liquids (e.g. [C_4C_1IM][Cl], [C_2C_1IM][Me_2PO_4]) was performed under heated conditions to guarantee the liquid state. In case of SC (solid catalyst) the thermal procedure was repeated but without impregnation of ionic liquids in-between. Synthesis repetitions demonstrated good reproducibility.

Catalyst characterization

Powder X-ray diffraction (XRD) patterns were collected in a Bragg-Brentano geometry on a Rigaku SmartLab diffractrometer with a rotating anode (9 kW, 45 kV, 200 mA, Cu-Kα λ = 1.541874 Å) radiation source and a HyPix-3000 multi-dimensional detector (1D mode). Samples were prepared on a quartz sample holder and data was collected in a 2θ range of 8 to 90 ° in steps of 0.01° and a scan rate of 0.5 °/min using a divergence slit of 0.125 °. Additional data was collected in the 2θ range of 1-50 ° in steps of 0.01 °, a scan rate of 5 °/min and a divergence slit of 0.02 °. Measured patterns were evaluated qualitatively by comparison with entries from the ICSD data base. Nitrogen physisorption was performed on a Micromeritics 3Flex at -196 °C. Samples were degassed at 80 °C (5 °C/min, 3 h) in vacuum (< $1.33 \cdot 10^{-2}$ mbar) prior to measurement. Brunauer-Emmett-Teller (BET) surface area was calculated with the Micromeritics MicroActive software (version 5.02) in the relative pressure range of 0.05 to 0.15. Pore volumes were deduced from the nitrogen quantity adsorbed at P/P^0 > 0.98. Pore size was estimated according to Barrett-Joyner-Halenda (BJH) algorithm on desorption isotherm (Harkins and Jura model with standard correction). Thermogravimetric analysis (TGA) was done on a Netzsch STA 449F3 instrument in the temperature range from 45 to 700 °C (5 °C/min, 40 mL/min air). Long-term stability was measured at 150, 200 and 250 °C (40 mL/min air) with a heating ramp of 10 °C/min and hold time of 5 h. Data analysis was done via the Netzsch Proteus – Thermal Analysis software (version 6.1.0). X-ray photoelectron spectroscopy (XPS) measurements were done on a custom spectrometer from SPECS GmbH with a

Phoibos 150 1D-DLD hemispherical energy analyzer and a monochromatic Al-Kα radiation source (E = 1486.6 eV, 15 kV, 200 W) in the medium area lens mode. Survey scans were performed at 50 eV pass energy while element-specific high-resolution scans were conducted at 20 eV pass energy. All measurements were obtained under $<1\cdot10^{-9}$ mbar vacuum using an electron flood gun and referenced to Pd $3d_{5/2}$ at 335.4 eV for charging correction. Fitting and deconvolution of the obtained data was performed with the CasaXPS software (version 2.3.25PR1.0). Ionic liquid modified samples were obtained by dip-coating cleaned palladium foil into a solution of the respective ionic liquid in acetone (1:100 v:v) or pure acetone for 10 s and dried for 1 h at 80 °C. Transmission electron microscopy (TEM) images were taken with a Hitachi H-7500 transmission electron microscope equipped with a LaB_6 cathode at an acceleration voltage of 100 kV. High-angle annular dark-field (HAADF) scanning transmission electron micrographs as well as energy dispersive x-ray mapping (EDX) was performed on a Thermo Scientific Talos F200x (scanning) transmission microscope equipped with a SuperX EDS system at an acceleration voltage of 200 kV. Samples were prepared by sprinkling the dry powder onto a 400 mesh carbon lacey copper TEM-grid. Elemental composition by EDX was investigated using a Hitachi TM3030 Plus table-top scanning electron microscope (SEM) equipped with an Oxford Instruments Xplore Compact 30 detector at an acceleration voltage of 15 kV. Samples for SEM measurements were prepared by sprinkling dry specimen on a sticky carbon tape. Inductively Coupled Plasma-Optical Emission Spectroscopy (ICP-OES) was performed on a Spectrogreen DSOI FMD46 instrument with UVPlus optics using commercial ICP standards for Pd and Ag (Bernd Kraft) for calibration.

Catalytic testing

Handling of pressurized acetylene requires several strict safety measures (e.g. explosion-safe cubicle, check valves, remote control to magnetic valves, purging possibilities, etc.) and should therefore not be performed in a common laboratory. For more information on safety issues when handling pressurized acetylene as well as further details about the reactor setup see the dissertation of I.-T. Trotus[1] or previously published studies[2]. Catalytic testing was conducted in a plug-flow fixed bed reactor (8 mm i.d.) under 10 bar pressure at temperatures between 60 and 160 °C. The reactor was heated via an external oven equipped with a thermocouple ($T_{heating}$). Temperature inside the catalyst bed was measured at up to four different positions (T top 1, T top 2, T middle, T bottom). Hydrogen, nitrogen, ethylene, acetylene and methane gas were purchased from Air Liquide. Acetylene gas was cleaned from acetone traces via a bed of alumina and zeolite A extrudates and compressed to 25 bar prior to use. All other gases were used without further purification. The flow rate of gases was controlled separately via previously calibrated mass flow controllers to a WHSV between 36 000 and 48 000 $cm^3\ h^{-1}\ g^{-1}_{cat}$. 200 mg of catalyst (mass of ionic liquids subtracted) was mixed with 3.0 g of silicon carbide as dilutant and placed onto a metal sieve inside the reactor. Quartz wool was added above to fix the position of the catalyst bed. All tubes downstream of the reactor were heated to 150 °C to avoid condensation of high-boiling oligomerisation products. Methane was added as internal standard to the product stream after the reactor. The composition of feed and product gas stream was analyzed via an online gas chromatograph (Agilent 7890B) equipped with three sequential columns (Rxi-5Sil MS, RT-alumina BOND/Na_2SO_4, RT-Msieve 5A) from Restek and two FID and one TCD detector. Flows of detected compounds (ethane, ethylene, acetylene, propane, propene, butane, 1-butene, 2-trans-butene, 2-cis-butene) were determined via the peak area ratio to methane, which was set to a constant flow, using response factors from previous calibration. The acetylene (or ethylene) conversion ($X_{C_2H_2}$) was calculated according to equation (1) as fraction of the molar flow of acetylene (or ethylene) leaving ($\dot{n}_{C_2H_2,\ out}$) over acetylene (or ethylene) fed into the reactor ($\dot{n}_{C_2H_2,\ in}$). Selectivity to ethylene, C_3 (propane, propene) and C_4 (butane, 1-butene, trans-2-butene, cis-2-butene) (S_{C_x}) were calculated via equation (2) using the difference between molar flow in the feed ($\dot{n}_{C_x,\ in}$, when present) and product gas stream ($\dot{n}_{C_x,\ out}$) over the molar flow of converted acetylene taking the number of carbon atoms of the compound (a_{C_x}) into account. Carbon balance (CB) was calculated via equation (3) using the molar flows of compounds in the product stream ($\dot{n}_{C_x,out}$) and in the feed ($\dot{n}_{C_x,in}$) multiplied with their number of carbon atoms (a_{C_x}). The selectivity to high-boiling hydrocarbons ($S_{C>4}$) was calculated indirectly via carbon missing in the carbon balance as shown in equation (4). Several catalytic tests were repeated multiple times to assess error bars (standard deviation) demonstrating good reproducibility. Standard deviation on selectivities was always smaller than ± 2.5% (abs.).

(1) $$X_{C_2H_2} = 1-\frac{\dot{n}_{C_2H_2,out}}{\dot{n}_{C_2H_2,in}}$$

(2) $$S_{C_x} = \frac{(\dot{n}_{C_x,out}-\dot{n}_{C_x,in})\cdot\left({}^{a_{C_x}}/_{2}\right)}{\dot{n}_{C_2H_2,in}-\dot{n}_{C_2H_2,out}}$$

(3) $$CB = \frac{\sum_{x=1}^{4}(a_{C_x}\cdot\dot{n}_{C_x,out})}{\sum_{x=1}^{4}(a_{C_x}\cdot\dot{n}_{C_x,in})}$$

(4) $$S_{C>4} = 100\%-\sum_{x=1}^{4}S_{C_x}$$

Tables

Table S1. Pore volume and BET surface area of silica gel, SC and corresponding SCILLs.

catalyst	pore volume (cm^3/g)	BET surface area (m^2/g)
silica gel	1.13	316
SC	1.12	302
$[C_4C_1IM][TFA]$-SCILL	< 0.01	2
$[C_4C_1IM][OAc]$-SCILL	< 0.01	2
$[C_4C_1IM][Cl]$-SCILL	< 0.01	< 1
$[C_4C_1IM][Me_2PO_4]$-SCILL	< 0.01	1
$[C_4C_1IM][MeSO_4]$-SCILL	0.02	3
$[C_4C_1IM][DCA]$-SCILL	< 0.01	1
$[C_4C_1IM][TCM]$-SCILL	< 0.01	1
$[C_4C_1IM][PF_6]$-SCILL	< 0.01	1
$[C_4C_1IM][BF_4]$-SCILL	< 0.01	1
$[C_4C_1IM][NTf_2]$-SCILL	< 0.01	1

Table S2. Calculated and experimental weight loss of SC and SCILLs via TGA (see Figure S18 for decomposition graph).

catalyst	calc. (w.-%)	exp. (w.-%)
SC	0	0.7
$[C_4C_1IM][TFA]$-SCILL	58.0	59.1
$[C_4C_1IM][OAc]$-SCILL	54.1	57.1
$[C_4C_1IM][Cl]$-SCILL	55.0	57.4
$[C_4C_1IM][Me_2PO_4]$-SCILL	56.5	51.5
$[C_4C_1IM][MeSO_4]$-SCILL	57.8	58.6
$[C_4C_1IM][DCA]$-SCILL	54.3	55.4
$[C_4C_1IM][TCM]$-SCILL	54.1	53.7
$[C_4C_1IM][PF_6]$-SCILL	60.5	64.1
$[C_4C_1IM][BF_4]$-SCILL	59.3	60.9
$[C_4C_1IM][NTf_2]$-SCILL	61.7	63.5

Table S3-1. Calculated and measured (experimental) elemental composition of SC and SCILLs via SEM-EDX.

catalyst	Si		O		Pd		N	
	calc.	exp.	calc.	exp.	calc.	exp.	calc.	exp.
SC	46.70	44.46	53.20	55.14	0.10	0.30	-	-
$[C_4C_1IM][TFA]$-SCILL	19.64	25.58	38.24	33.33	0.04	0.15	13.89	7.97
$[C_4C_1IM][OAc]$-SCILL	21.46	35.66	53.27	52.26	0.05	0.24	25.23	11.71
$[C_4C_1IM][Cl]$-SCILL	21.03	30.96	23.96	39.36	0.05	0.18	24.27	12.80
$[C_4C_1IM][Me_2PO_4]$-SCILL	20.31	28.74	52.54	50.16	0.04	0.27	12.87	9.30
$[C_4C_1IM][MeSO_4]$-SCILL	19.73	28.15	52.26	50.67	0.04	0.25	13.04	9.25
$[C_4C_1IM][DCA]$-SCILL	21.35	34.28	24.32	39.71	0.05	0.23	54.28	24.91
$[C_4C_1IM][TCM]$-SCILL	21.46	37.54	24.45	39.05	0.05	0.26	54.04	23.03
$[C_4C_1IM][PF_6]$-SCILL	18.43	19.63	20.99	17.49	0.04	0.22	9.80	8.20
$[C_4C_1IM][BF_4]$-SCILL	19.01	25.94	21.66	23.88	0.04	0.24	15.97	9.23
$[C_4C_1IM][NTf_2]$-SCILL	17.87	17.85	34.26	29.15	0.04	0.10	9.13	8.63

Table S3-2. Calculated and measured (experimental) elemental composition of SC and SCILLs via SEM-EDX.

catalyst	S		P		F		Cl	
	calc.	exp.	calc.	exp.	calc.	exp.	calc.	exp.
SC	-	-	-	-	-	-	-	-
$[C_4C_1IM][TFA]$-SCILL	-	-	-	-	28.25	29.09	-	-
$[C_4C_1IM][OAc]$-SCILL	-	-	-	-	-	-	-	-
$[C_4C_1IM][Cl]$-SCILL	-	-	-	-	-	-	30.71	16.7
$[C_4C_1IM][Me_2PO_4]$-SCILL	-	-	14.23	11.45	-	-	-	-
$[C_4C_1IM][MeSO_4]$-SCILL	14.92	11.68	-	-	-	-	-	-
$[C_4C_1IM][DCA]$-SCILL	-	-	-	-	-	-	-	0.15
$[C_4C_1IM][TCM]$-SCILL	-	-	-	-	-	-	-	-
$[C_4C_1IM][PF_6]$-SCILL	-	-	-	-	39.89	44.40	-	-
$[C_4C_1IM][BF_4]$-SCILL	-	-	-	-	43.32	40.47	-	-
$[C_4C_1IM][NTf_2]$-SCILL	13.93	13.31	-	-	24.76	30.83	-	-

Table S3-3. Calculated and measured (experimental) elemental composition of SC and SCILLs via SEM-EDX.

catalyst	Na		K	
	calc.	exp.	calc.	exp.
SC	-	0.10	-	-
$[C_4C_1IM][TFA]$-SCILL	-	0.12	-	3.77
$[C_4C_1IM][OAc]$-SCILL	-	0.11	-	-
$[C_4C_1IM][Cl]$-SCILL	-	-	-	-
$[C_4C_1IM][Me_2PO_4]$-SCILL	-	0.07	-	-
$[C_4C_1IM][MeSO_4]$-SCILL	-	-	-	-
$[C_4C_1IM][DCA]$-SCILL	-	0.73	-	-
$[C_4C_1IM][TCM]$-SCILL	-	0.13	-	-
$[C_4C_1IM][PF_6]$-SCILL	-	-	-	-
$[C_4C_1IM][BF_4]$-SCILL	-	0.23	-	-
$[C_4C_1IM][NTf_2]$-SCILL	-	0.13	-	-

Table S4. Temperature of 50% decomposition ($T_{dec, 50\%}$) and mass loss at isothermal conditions (150, 200 and 250 °C for 5 h) of studied ionic liquids via TGA (Figures S19-21).

compound	$T_{dec, 50\%}$ (°C)	mass loss (150 °C, w.-%)	mass loss (200 °C, w.-%)	mass loss (250 °C, w.-%)
$[C_4C_1IM][TFA]$	191	84.4	90.6	95.7
$[C_4C_1IM][OAc]$	226	-	-	-
$[C_4C_1IM][Cl]$	262	-	-	-
$[C_4C_1IM][Me_2PO_4]$	289	4.8	29.3	66.8
$[C_4C_1IM][MeSO_4]$	320	7.8	9.9	19.2
$[C_4C_1IM][DCA]$	359	2.8	7.0	49.5
$[C_4C_1IM][TCM]$	371	-	-	-
$[C_4C_1IM][PF_6]$	397	1.3	5.1	32.4
$[C_4C_1IM][BF_4]$	414	-	-	-
$[C_4C_1IM][NTf_2]$	426	<0.1	0.9	11.5

Table S5-1. Selectivity performance of SC and SCILLs averaged over 20 hours on-stream (Figure 2) and summarized properties of ionic liquids from literature.

SC/SCILL catalyst	S C_2H_4 (%)	S C_2H_6 (%)	S $C_{>2}$ (%)	$\alpha_{C2H2/C2H4}$ (298-323 K)	$H_{v,C2H2}$ (MPa)[a]	$H_{v,C2H4}$ (MPa)[a]	$H_{v,H2}$ (MPa)[b]
$[MeSO_4]^-$	82	7	12	20[3]	1.09[3]	21.79[3]	168.4[4]
$[DCA]^-$	80	7	13	(9)[5]	(1.84)[5]	(16.22)[5]	-
$[TCM]^-$	78	7	15	-	-	-	-
$[PF_6]^-$	78	8	14	11[6]	2.22[7]	25.27[c]	660[8]
$[Me_2PO_4]^-$	77	13	11	27[3]	0.49[3]	13.42[3]	-
$[Cl]^-$	74	8	18	12[9]	-	-	-
$[BF_4]^-$	70	9	21	11[3]	1.75[3]	19.22[3]	580[8]
$[TFA]^-$	69	14	18	10[3]	1.42[3]	13.63[3]	490[8]
$[OAc]^-$	68	12	20	28[3]	0.64[3]	17.59[3]	-
$[NTf_2]^-$	58	12	30	4[3]	2.18[3]	8.29[3]	450[8]
SC	-100	175	25	-	-	-	-

[a] at 298-313 K, 1 atm, [b] at 293-313 K, 1 atm, ( ) values estimated from $[C_4C_1Pyrr][X]$,
[c] calculated from $\alpha_{C2H2/C2H4}$ and $H_{v,C2H2}$

Table S5-2. Selectivity performance of SC and SCILLs averaged over 20 hours on-stream (Figure 2) and summarized properties of ionic liquids from literature.

SC/SCILL catalyst	S C_2H_4 (%)	S C_2H_6 (%)	S $C_{>2}$ (%)	viscosity (mPa·s)[a]	Hydrogen bond bascisity β	ion volume (nm^3)
$[MeSO_4]^-$	82	7	12	163[10]	0.75[11]	0.107[12]
$[DCA]^-$	80	7	13	33[10]	0.64[11]	0.089[12]
$[TCM]^-$	78	7	15	26[10]	0.54[11]	0.134[13]
$[PF_6]^-$	78	8	14	267[10]	0.44[11]	0.111[12]
$[Me_2PO_4]^-$	77	13	11	534[10]	1.12[11]	0.145[12]
$[Cl]^-$	74	8	18	40890[14]	0.95[11]	0.047[12]
$[BF_4]^-$	70	9	21	104[10]	0.55[11]	0.079[12]
$[TFA]^-$	69	14	18	~70[15]	0.74[11]	0.103[12]
$[OAc]^-$	68	12	20	208[10]	1.20[11]	0.078[12]
$[NTf_2]^-$	58	12	30	49[10]	0.42[11]	0.230[12]
SC	-100	175	25	-	-	-

[a] at 293-298 K

Table S6. Pore volume and BET surface area of SC and 0 to 161 w.-% $[NTf_2]^-$-SCILL (Figure 3b).

catalyst	pore volume (cm^3/g)	BET surface area (m^2/g)
SC	1.12	302
40w.-% $[C_4C_1IM][NTf_2]$-SCILL	0.59	132
80w.-% $[C_4C_1IM][NTf_2]$-SCILL	0.31	67
120w.-% $[C_4C_1IM][NTf_2]$-SCILL	0.13	28
161w.-% $[C_4C_1IM][NTf_2]$-SCILL	< 0.01	1

Table S7. Calculated and experimental weight loss of SC and 0 to 161 w.-% $[NTf_2]^-$-SCILL (Figure 3b) via TGA.

catalyst	calc. (w.-%)	exp. (w.-%)
SC	0	0.7
40w.-% $[C_4C_1IM][NTf_2]$-SCILL	28.6	29.4
80w.-% $[C_4C_1IM][NTf_2]$-SCILL	44.4	44.7
120w.-% $[C_4C_1IM][NTf_2]$-SCILL	54.5	57.0
161w.-% $[C_4C_1IM][NTf_2]$-SCILL	61.7	63.5

Table S8-1. Calculated and measured elemental composition of SC and 0 to 161 w.-% $[NTf_2]^-$-SCILL (Figure 3b) via SEM-EDX.

catalyst	Si		O		Pd		N	
	calc.	exp.	calc.	exp.	calc.	exp.	calc.	exp.
SC	46.70	44.46	53.20	55.14	0.10	0.30	-	-
40w.-% $[C_4C_1IM][NTf_2]$-SCILL	33.36	36.30	44.44	44.32	0.07	0.19	4.23	3.87
80w.-% $[C_4C_1IM][NTf_2]$-SCILL	25.94	30.47	39.57	39.50	0.06	0.18	6.57	5.36
120w.-% $[C_4C_1IM][NTf_2]$-SCILL	21.23	27.38	36.47	36.91	0.05	0.24	8.07	6.03
161w.-% $[C_4C_1IM][NTf_2]$-SCILL	17.87	17.85	34.26	29.15	0.04	0.10	9.13	8.63

Table S8-2. Calculated and measured elemental composition of SC and 0 to 161 w.-% $[NTf_2]^-$-SCILL (Figure 3b) via SEM-EDX.

catalyst	S		P		F		Cl	
	calc.	exp.	calc.	exp.	calc.	exp.	calc.	exp.
SC	-	-	-	-	-	-	-	-
40w.-% $[C_4C_1IM][NTf_2]$-SCILL	6.45	4.75	-	-	11.46	10.56	-	-
80w.-% $[C_4C_1IM][NTf_2]$-SCILL	10.03	7.66	-	-	17.83	16.71	-	-
120w.-% $[C_4C_1IM][NTf_2]$-SCILL	12.31	9.82	-	-	21.88	19.50	-	-
161w.-% $[C_4C_1IM][NTf_2]$-SCILL	13.93	13.31	-	-	24.76	30.83	-	-

Table S8-3. Calculated and measured elemental composition of SC and 0 to 161 w.-% $[NTf_2]^-$-SCILL (Figure 3b) via SEM-EDX.

catalyst	Na		K	
	calc.	exp.	calc.	exp.
SC	-	0.10	-	-
40w.-% $[C_4C_1IM][NTf_2]$-SCILL	-	-	-	
80w.-% $[C_4C_1IM][NTf_2]$-SCILL	-	0.12	-	-
120w.-% $[C_4C_1IM][NTf_2]$-SCILL	-	0.11	-	-
161w.-% $[C_4C_1IM][NTf_2]$-SCILL	-	0.13	-	-

Table S9. Pore volume and BET surface area of SC and $[C_xC_1IM][Me_2PO_4]$-SCILLs (Figure S10 and S47-49).

catalyst	pore volume (cm^3/g)	BET surface area (m^2/g)
SC	1.12	302
$[C_4C_1IM][Me_2PO_4]$-SCILL	< 0.01	1
$[C_2C_1IM][Me_2PO_4]$-SCILL	0.02	3
$[C_1C_1IM][Me_2PO_4]$-SCILL	< 0.01	2

Table S10. Calculated and experimental weight loss of SC and $[C_xC_1IM][Me_2PO_4]$-SCILLs via TGA (Figure S10 and S47-49).

catalyst	calc.	exp.
	(w.-%)	(w.-%)
SC	0	0.7
$[C_4C_1IM][Me_2PO_4]$-SCILL	56.5	51.5
$[C_2C_1IM][Me_2PO_4]$-SCILL	57.7	50.9
$[C_1C_1IM][Me_2PO_4]$-SCILL	56.3	51.1

Table S11-1. Calculated and measured (experimental) elemental composition of SC and $[C_xC_1IM][Me_2PO_4]$-SCILLs via SEM-EDX (Figure S10 and S47-49).

catalyst	Si		O		Pd		N	
	calc.	exp.	calc.	exp.	calc.	exp.	calc.	exp.
SC	46.70	44.46	53.20	55.14	0.10	0.30	-	-
$[C_4C_1IM][Me_2PO_4]$-SCILL	20.31	28.74	52.54	50.16	0.04	0.27	12.87	9.30
$[C_2C_1IM][Me_2PO_4]$-SCILL	19.73	25.21	52.53	51.66	0.04	0.23	13.15	10.71
$[C_1C_1IM][Me_2PO_4]$-SCILL	20.41	25.90	52.55	51.58	0.04	0.26	12.82	10.09

Table S11-2. Calculated and measured (experimental) elemental composition of SC and $[C_xC_1IM][Me_2PO_4]$-SCILLs via SEM-EDX (Figure S10 and S47-49).

catalyst	S		P		F		Cl	
	calc.	exp.	calc.	exp.	calc.	exp.	calc.	exp.
SC	-	-	-	-	-	-	-	-
$[C_4C_1IM][Me_2PO_4]$-SCILL	-	-	14.23	11.45	-	-	-	-
$[C_2C_1IM][Me_2PO_4]$-SCILL	-	-	14.54	12.20	-	-	-	-
$[C_1C_1IM][Me_2PO_4]$-SCILL	-	-	14.18	11.78	-	0.39	-	-

Table S11-3. Calculated and measured (experimental) elemental composition of SC and $[C_xC_1IM][Me_2PO_4]$-SCILLs via SEM-EDX (Figure S10 and S47-49).

catalyst	Na		K	
	calc.	exp.	calc.	exp.
SC	-	0.10	-	-
$[C_4C_1IM][Me_2PO_4]$-SCILL	-	0.07	-	-
$[C_2C_1IM][Me_2PO_4]$-SCILL	-	-	-	-
$[C_1C_1IM][Me_2PO_4]$-SCILL	-	-	-	-

Table S12. Temperature of 50% decomposition ($T_{dec, 50\%}$) and mass loss at isothermal conditions (150, 200 and 250 °C for 5 h) of $[C_xC_1IM][Me_2PO_4]$ ionic liquids via TGA.

compound	$T_{dec, 50\%}$ (°C)	mass loss (150 °C, w.-%)	mass loss (200 °C, w.-%)	mass loss (250 °C, w.-%)
$[C_4C_1IM][Me_2PO_4]$	289	4.8	29.3	66.8
$[C_2C_1IM][Me_2PO_4]$	283	-	-	-
$[C_1C_1IM][Me_2PO_4]$	289	6.3	-	-

Table S13-1. Selectivity performance of $[C_xC_1IM][Me_2PO_4]$-SCILLs averaged over 0-2 hours on-stream (Figure S10 and S47-49) and summarized properties of ionic liquids from literature.

SC/SCILL catalyst	S C_2H_4 (%)	S C_2H_6 (%)	S $C_{>2}$ (%)	$\alpha_{C2H2/C2H4}$ (298-323 K)	$H_{v,C2H2}$ (MPa)[a]	$H_{v,C2H4}$ (MPa)[a]	$H_{v,H2}$ (MPa)[b]
$[C_1C_1IM]^+$	79	9	12	41[3]	0.64[3, 7]	26.16[3]	-
$[C_2C_1IM]^+$	78	10	12	-	-	-	-
$[C_4C_1IM]^+$	76	14	10	27[3]	0.49[3]	13.42[3]	-

[a] at 298-313 K, 1 atm, [b] at 293-313 K, 1 atm

Table S13-2. Selectivity performance of [C_xC_1IM][Me_2PO_4]-SCILLs averaged over 0-2 hours on-stream (Figure S10 and S47-49) and summarized properties of ionic liquids from literature.

SC/SCILL catalyst	S C_2H_4 (%)	S C_2H_6 (%)	S $C_{>2}$ (%)	viscosity (mPa·s)[a]	Hydrogen bond bascisity β	ion volume (nm^3)
[C_1C_1IM]$^+$	79	9	12	437[10]	-	0.129[12]
[C_2C_1IM]$^+$	78	10	12	-	-	0.152[12]
[C_4C_1IM]$^+$	76	14	10	534[10]	1.12[11]	0.198[12]

[a] at 293-298 K

Figures

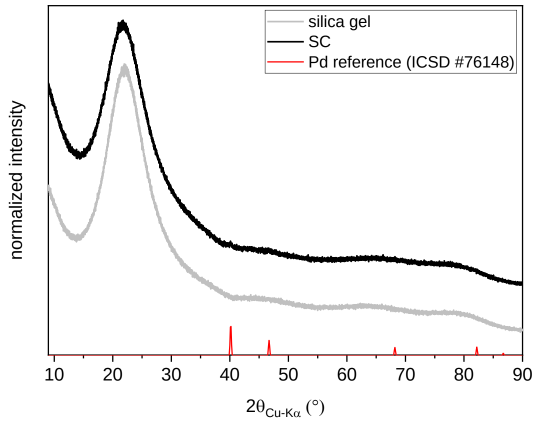

Figure S1. XRD pattern of silica gel and SC with ICSD reference for Pd.

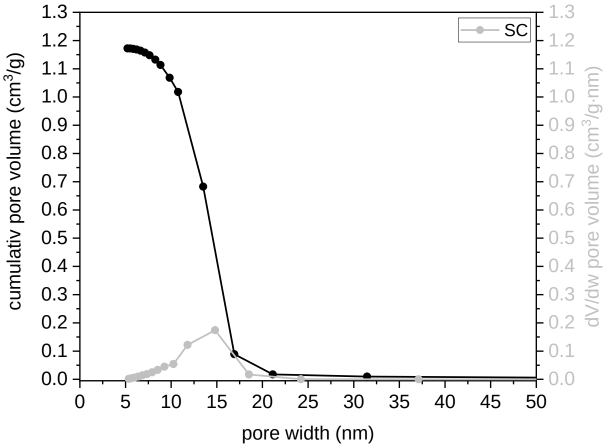

Figure S2. Pore size distribution of SC via cumulative and incremental pore volume change.

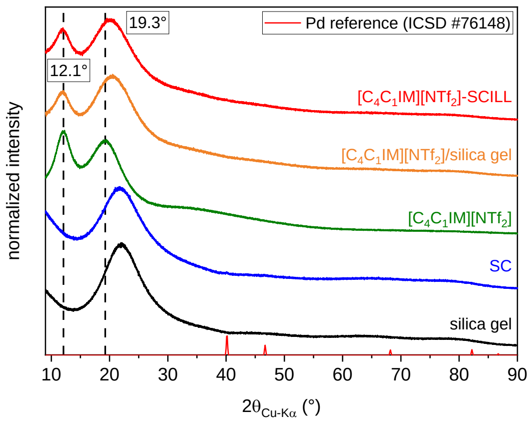

Figure S3. XRD pattern of silica gel, SC, [C_4C_1IM][NTf_2], [NTf_2]$^-$-SCILL and [C_4C_1IM][NTf_2]-silica gel revealing reflections resulting from the ordering of cations and anions in the ionic liquid phase.

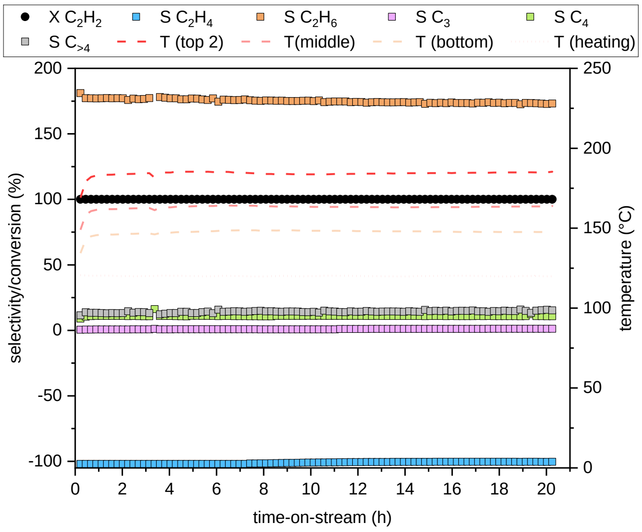

Figure S4. Ethylene, ethane, C_3, C_4, $C_{>4}$ selectivity and acetylene conversion of SC ("none", Figure 2) as well as temperature of heating and at different positions in the catalyst bed (top 1, top 2, middle, bottom) versus time-on-stream in the selective hydrogenation of concentrated acetylene streams (C_2H_2/C_2H_4/H_2 1:1:5, 10 bar, 120 °C, X_{C2H2} = 100%, CB ≥ 0.93, WHSV 42 000 cm^3 h^{-1} g^{-1}_{cat}).

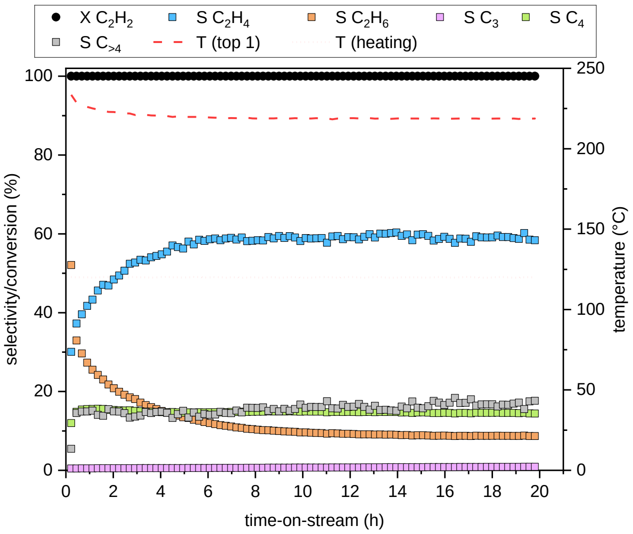

Figure S5. Ethylene, ethane, C_3, C_4, $C_{>4}$ selectivity and acetylene conversion of $[NTf_2]^-$-SCILL (Figure 2) as well as temperature of heating and at different positions in the catalyst bed (top 1, top 2, middle, bottom) versus time-on-stream in the selective hydrogenation of concentrated acetylene streams ($C_2H_2/C_2H_4/H_2$ 1:1:5, 10 bar, 120 °C, X_{C2H2} = 100%, CB ≥ 0.93, WHSV 42 000 cm^3 h^{-1} $g^{-1}{}_{cat}$).

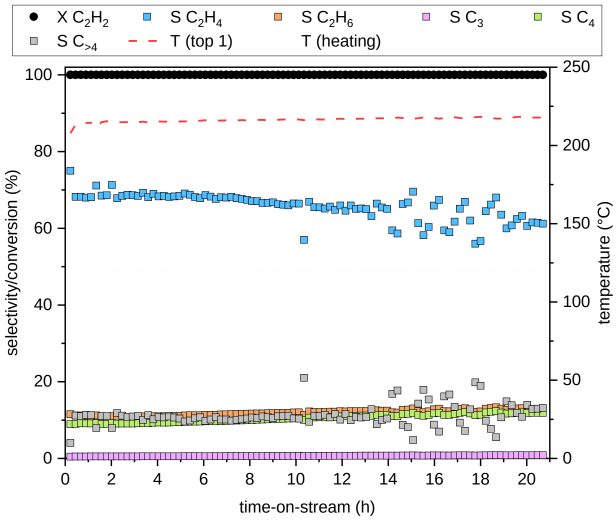

Figure S6. Ethylene, ethane, C_3, C_4 , $C_{>4}$ selectivity and acetylene conversion of $[OAc]^-$-SCILL (Figure 2) as well as temperature of heating and at different positions in the catalyst bed (top 1, top 2, middle, bottom) versus time-on-stream in the selective hydrogenation of concentrated acetylene streams ($C_2H_2/C_2H_4/H_2$ 1:1:5, 10 bar, 120 °C, X_{C2H2} = 100%, CB ≥ 0.96, WHSV 42 000 cm^3 h^{-1} $g^{-1}{}_{cat}$).

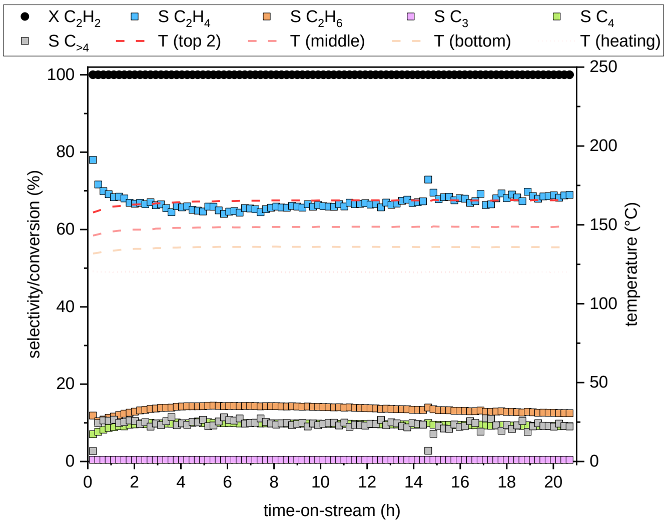

Figure S7. Ethylene, ethane, C_3, C_4 , $C_{>4}$ selectivity and acetylene conversion of $[TFA]^-$-SCILL (Figure 2) as well as temperature of heating and at different positions in the catalyst bed (top 1, top 2, middle, bottom) versus time-on-stream in the selective hydrogenation of concentrated acetylene streams ($C_2H_2/C_2H_4/H_2$ 1:1:5, 10 bar, 120 °C, X_{C2H2} = 100%, CB ≥ 0.96, WHSV 42 000 cm^3 h^{-1} g^{-1}_{cat}).

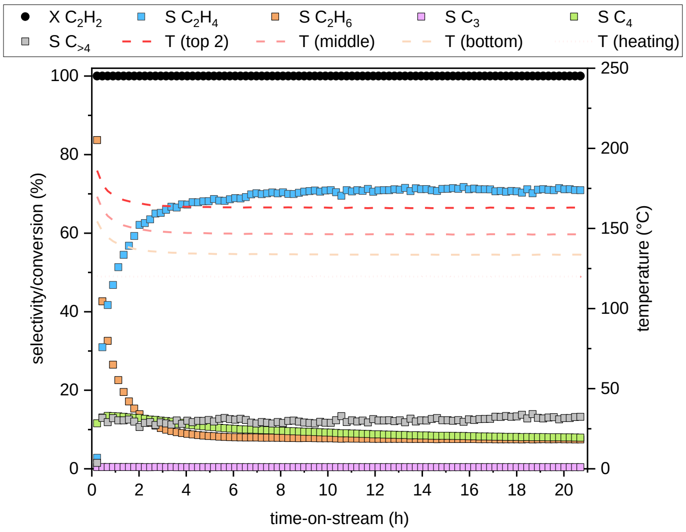

Figure S8. Ethylene, ethane, C_3, C_4 , $C_{>4}$ selectivity and acetylene conversion of $[BF_4]^-$-SCILL (Figure 2) as well as temperature of heating and at different positions in the catalyst bed (top 1, top 2, middle, bottom) versus time-on-stream in the selective hydrogenation of concentrated acetylene streams ($C_2H_2/C_2H_4/H_2$ 1:1:5, 10 bar, 120 °C, X_{C2H2} = 100%, CB ≥ 0.95, WHSV 42 000 cm^3 h^{-1} g^{-1}_{cat}).

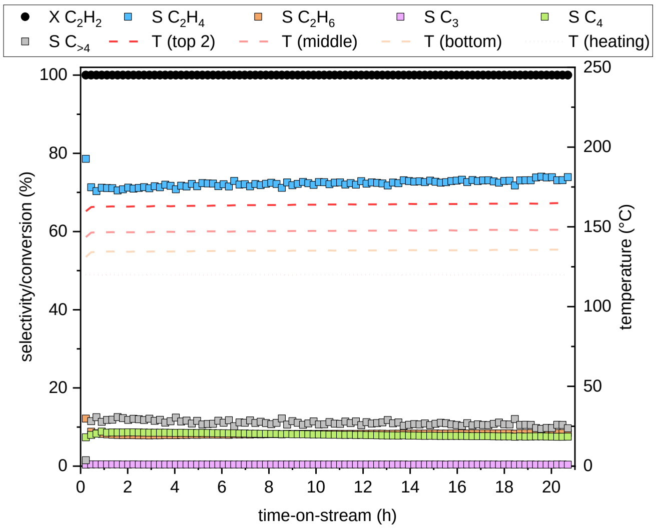

Figure S9. Ethylene, ethane, C_3, C_4 , $C_{>4}$ selectivity and acetylene conversion of [Cl]$^-$-SCILL (Figure 2) as well as temperature of heating and at different positions in the catalyst bed (top 1, top 2, middle, bottom) versus time-on-stream in the selective hydrogenation of concentrated acetylene streams ($C_2H_2/C_2H_4/H_2$ 1:1:5, 10 bar, 120 °C, X_{C2H2} = 100%, CB ≥ 0.95, WHSV 42 000 $cm^3\ h^{-1}\ g^{-1}_{cat}$).

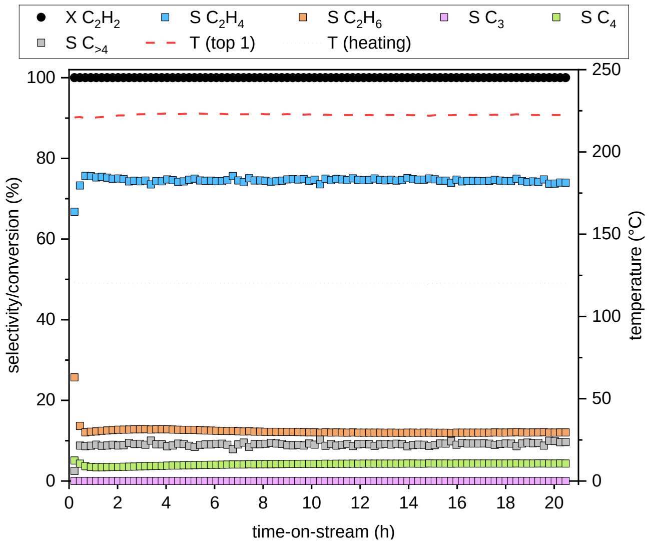

Figure S10. Ethylene, ethane, C_3, C_4 , $C_{>4}$ selectivity and acetylene conversion of [Me_2PO_4]$^-$-SCILL (Figure 2) as well as temperature of heating and at different positions in the catalyst bed (top 1, top 2, middle, bottom) versus time-on-stream in the selective hydrogenation of concentrated acetylene streams ($C_2H_2/C_2H_4/H_2$ 1:1:5, 10 bar, 120 °C, X_{C2H2} = 100%, CB ≥ 0.97, WHSV 42 000 $cm^3\ h^{-1}\ g^{-1}_{cat}$).

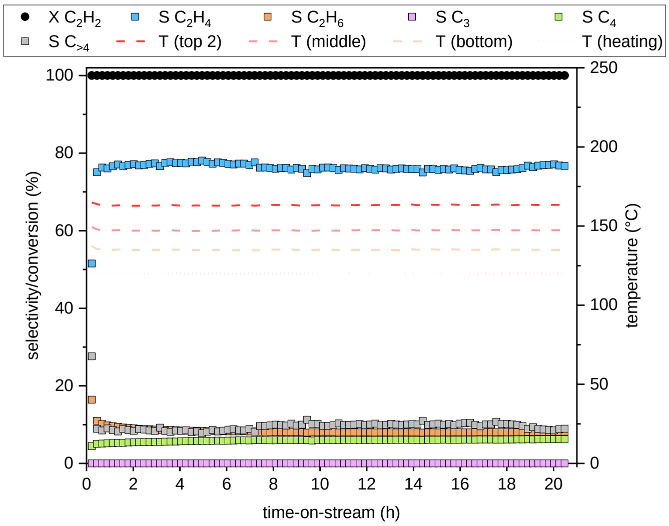

Figure S11. Ethylene, ethane, C_3, C_4 , $C_{>4}$ selectivity and acetylene conversion of $[PF_6]^-$-SCILL (Figure 2) as well as temperature of heating and at different positions in the catalyst bed (top 1, top 2, middle, bottom) versus time-on-stream in the selective hydrogenation of concentrated acetylene streams ($C_2H_2/C_2H_4/H_2$ 1:1:5, 10 bar, 120 °C, X_{C2H2} = 100%, CB ≥ 0.96, WHSV 42 000 $cm^3\ h^{-1}\ g^{-1}_{cat}$).

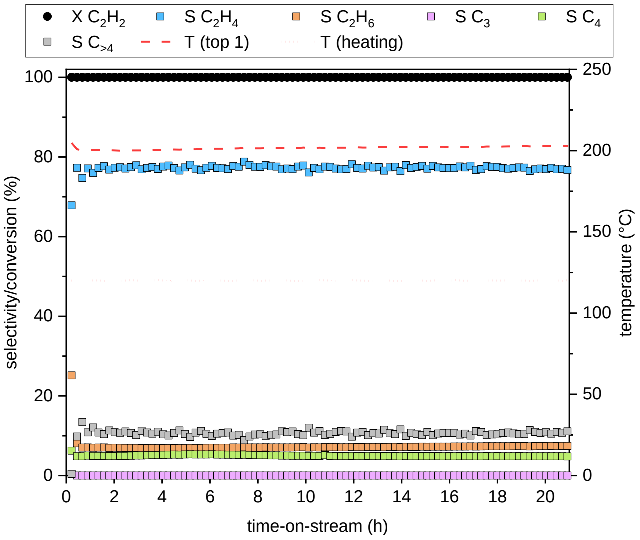

Figure S12. Ethylene, ethane, C_3, C_4 , $C_{>4}$ selectivity and acetylene conversion of $[TCM]^-$-SCILL (Figure 2) as well as temperature of heating and at different positions in the catalyst bed (top 1, top 2, middle, bottom) versus time-on-stream in the selective hydrogenation of concentrated acetylene streams ($C_2H_2/C_2H_4/H_2$ 1:1:5, 10 bar, 120 °C, X_{C2H2} = 100%, CB ≥ 0.95, WHSV 42 000 $cm^3\ h^{-1}\ g^{-1}_{cat}$).

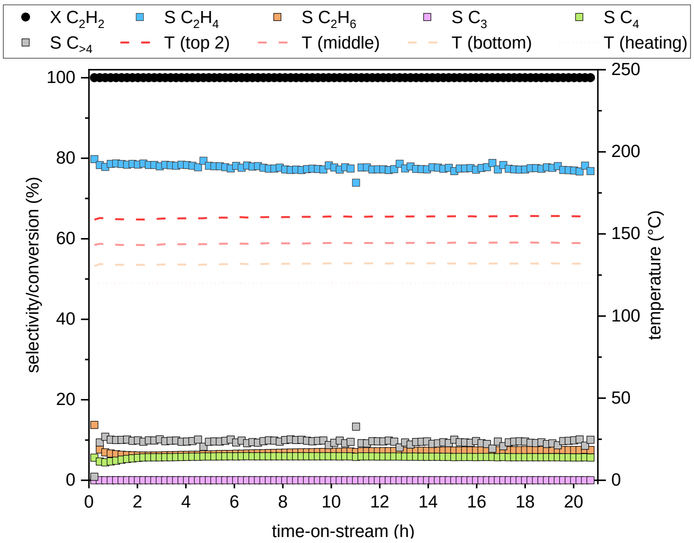

Figure S13. Ethylene, ethane, C_3, C_4 , $C_{>4}$ selectivity and acetylene conversion of $[DCA]^-$-SCILL (Figure 2) as well as temperature of heating and at different positions in the catalyst bed (top 1, top 2, middle, bottom) versus time-on-stream in the selective hydrogenation of concentrated acetylene streams ($C_2H_2/C_2H_4/H_2$ 1:1:5, 10 bar, 120 °C, X_{C2H2} = 100%, CB ≥ 0.96, WHSV 42 000 $cm^3\ h^{-1}\ g^{-1}_{cat}$).

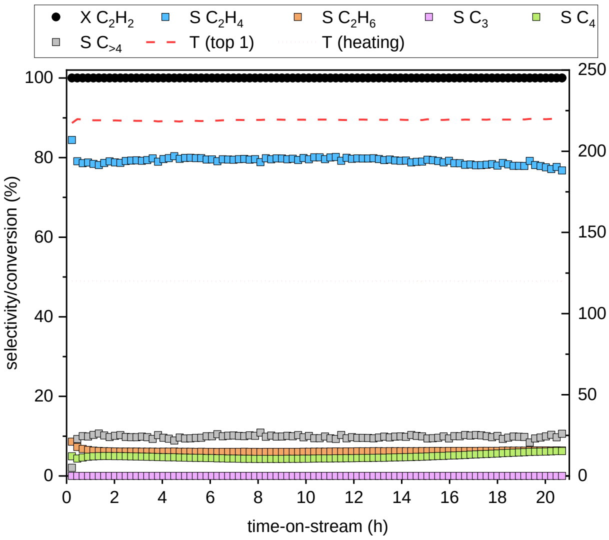

Figure S14. Ethylene, ethane, C_3, C_4 , $C_{>4}$ selectivity and acetylene conversion of $[MeSO_4]^-$-SCILL (Figure 2) as well as temperature of heating and at different positions in the catalyst bed (top 1, top 2, middle, bottom) versus time-on-stream in the selective hydrogenation of concentrated acetylene streams ($C_2H_2/C_2H_4/H_2$ 1:1:5, 10 bar, 120 °C, X_{C2H2} = 100%, CB ≥ 0.97, WHSV 42 000 $cm^3\ h^{-1}\ g^{-1}_{cat}$).

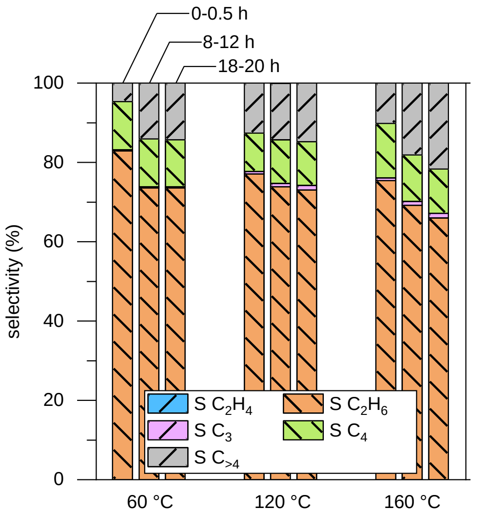

Figure S15. Average ethylene, ethane, C_3, C_4 and $C_{>4}$ selectivity of SC at different time-on-stream intervals (0-0.5 h, 8-12 h and 18-20 h) and temperatures (60, 120 and 160 °C) in the selective hydrogenation of concentrated acetylene streams ($C_2H_2/C_2H_4/H_2$ 1:1:5, 10 bar, X_{C2H2} = 100%, CB ≥ 0.93, WHSV 42 000 cm^3 h^{-1} g$^{-1}_{cat}$).

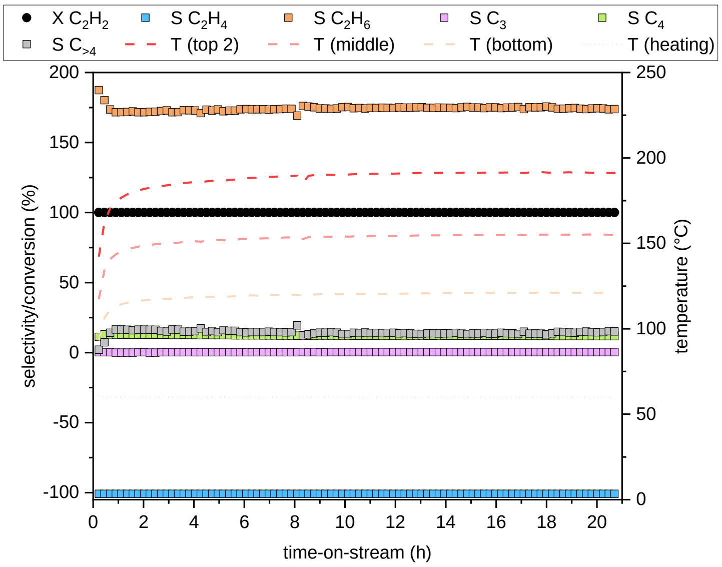

Figure S16. Ethylene, ethane, C_3, C_4 , $C_{>4}$ selectivity and acetylene conversion of SC as well as temperature of heating and at different positions in the catalyst bed (top 1, top 2, middle, bottom) versus time-on-stream in the selective hydrogenation of concentrated acetylene streams ($C_2H_2/C_2H_4/H_2$ 1:1:5, 10 bar, 60 °C, X_{C2H2} = 100%, CB ≥ 0.93, WHSV 42 000 cm^3 h^{-1} g$^{-1}_{cat}$).

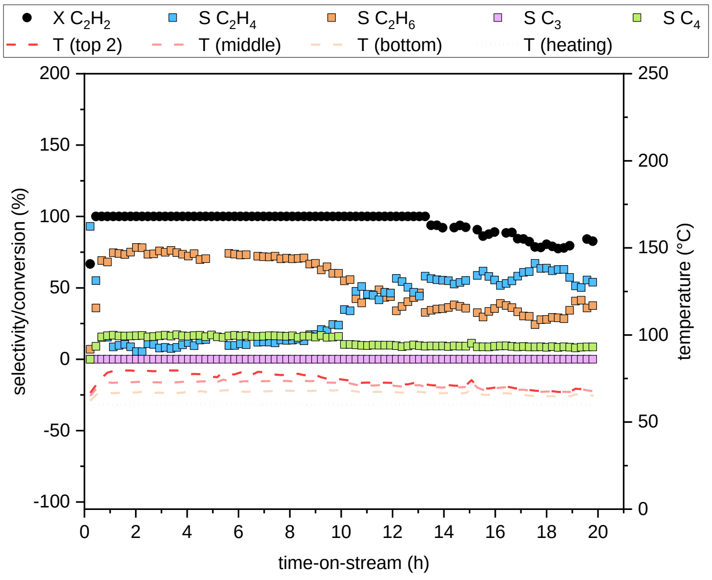

Figure S17. Ethylene, ethane, C_3, C_4 selectivity and acetylene conversion of SC as well as temperature of heating and at different positions in the catalyst bed (top 1, top 2, middle, bottom) versus time-on-stream in the selective hydrogenation of diluted acetylene streams ($C_2H_2/C_2H_4/H_2/N_2$ 1:50:1.5:18, 10 bar, 60 °C, X_{C2H2} = 100%, CB ≥ 0.97, WHSV 42 000 cm^3 h^{-1} g^{-1}_{cat}). Ethylene selectivity was in this reaction exceptionally calculated over closing the carbon balance (analogously to $C_{>4}$ in other reactions) with ethane, sum C_3 and sum C_4 due to the high ethylene feed concentration.

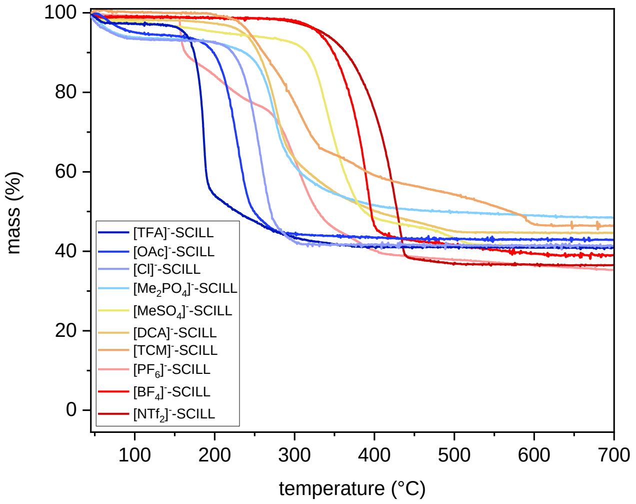

Figure S18. TGA decomposition curves of $[C_4C_1IM]^+$ based SCILLs at 5 °C/min heating rate and 40 mL/min air.

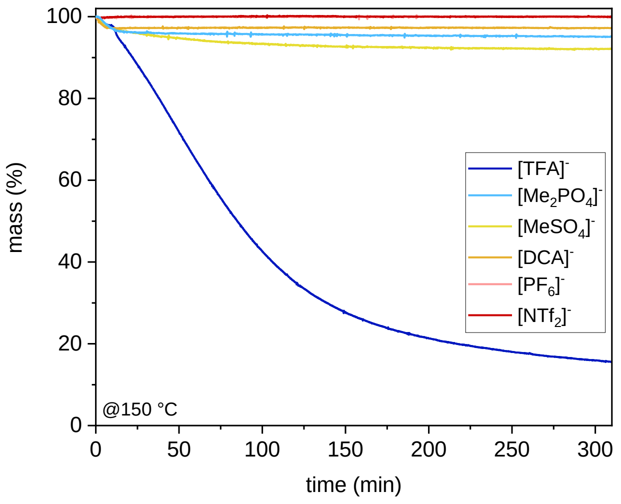

Figure S19. TGA decomposition curves of $[C_4C_1IM]^+$ based ionic liquids with different anions for long-term stability tests for 5 h at constant temperature of 150 °C (previous heating ramp 10 °C/min, 40 mL/min air).

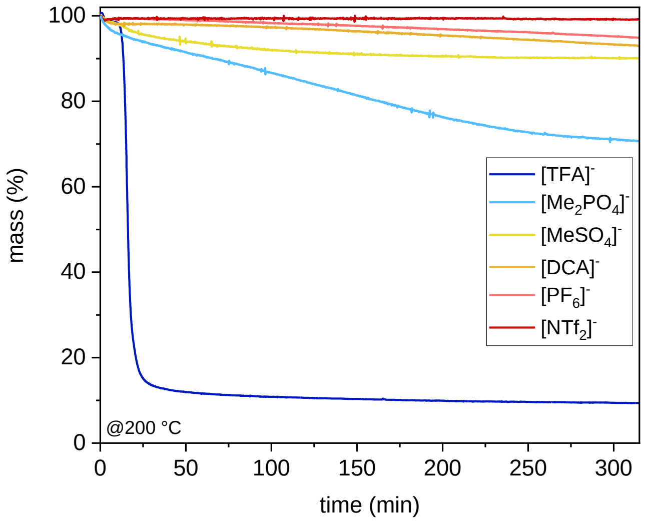

Figure S20. TGA decomposition curves of $[C_4C_1IM]^+$ based ionic liquids with different anions for long-term stability tests for 5 h at constant temperature of 200 °C (previous heating ramp 10 °C/min, 40 mL/min air).

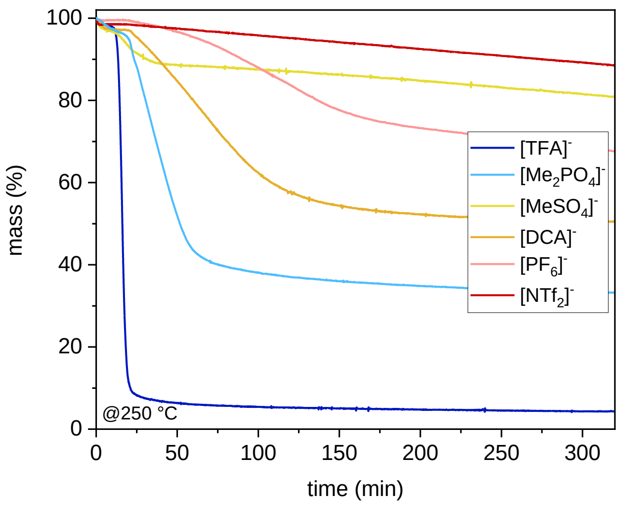

Figure S21. TGA decomposition curves of $[C_4C_1IM]^+$ based ionic liquids with different anions for long-term stability tests for 5 h at constant temperature of 250 °C (previous heating ramp 10 °C/min, 40 mL/min air).

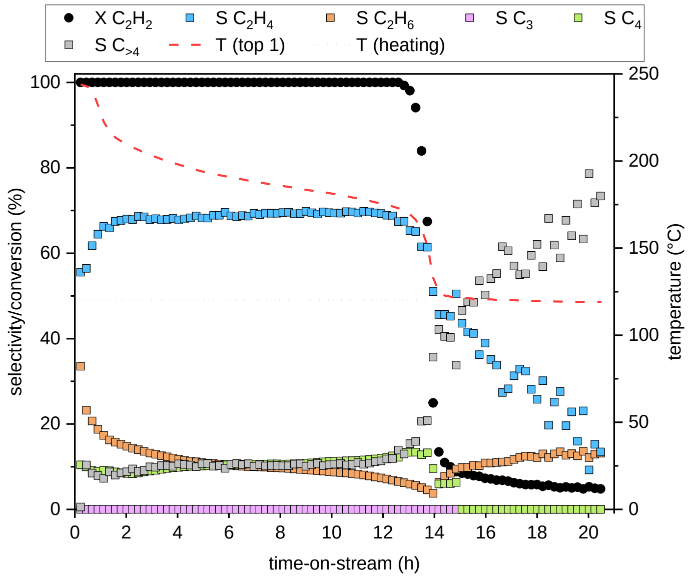

Figure S22. Ethylene, ethane, C_3, C_4 , $C_{>4}$ selectivity and acetylene conversion of 1 w.-% Pd_1Ag_9/silica gel as well as temperature of heating and at different positions in the catalyst bed (top 1, top 2, middle, bottom) versus time-on-stream in the selective hydrogenation of concentrated acetylene streams ($C_2H_2/C_2H_4/H_2$ 1:1:5, 10 bar, 120 °C, X_{C2H2} = 100%, CB ≥ 0.95, WHSV 42 000 cm^3 h^{-1} g^{-1}_{cat}).

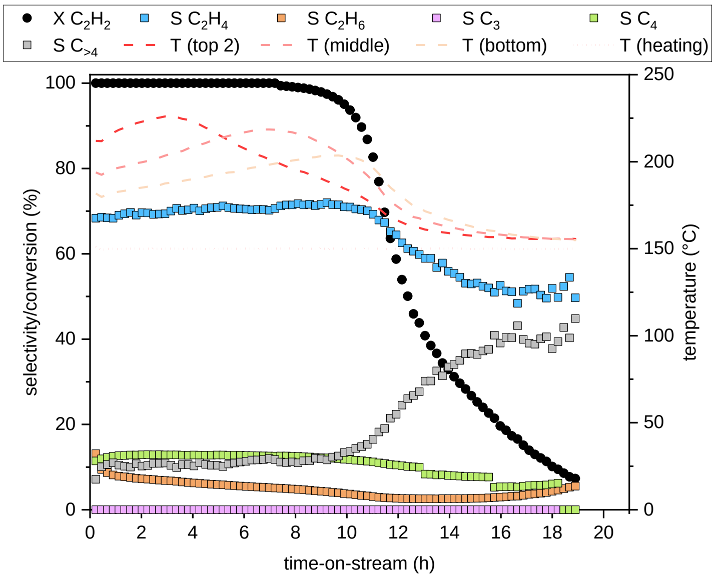

Figure S23. Ethylene, ethane, C_3, C_4 , $C_{>4}$ selectivity and acetylene conversion of 1 w.-% Pd_1Ag_9/zeolite Y as well as temperature of heating and at different positions in the catalyst bed (top 1, top 2, middle, bottom) versus time-on-stream in the selective hydrogenation of concentrated acetylene streams ($C_2H_2/C_2H_4/H_2$ 1:1:5, 10 bar, 150 °C, X_{C2H2} = 100%, CB ≥ 0.95, WHSV 48 000 cm^3 h^{-1} g^{-1}_{cat}, 175 mg catalyst) showing the migration of the hotspot in catalyst bed with deactivation.

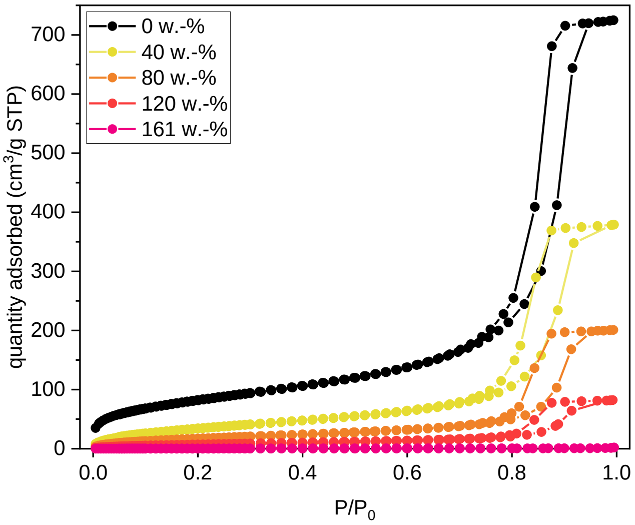

Figure S24. N_2 physisorption isotherms of SC and 40-161 w.-% $[NTf_2]^-$-SCILLs showing the stepwise pore filling of the silica gel support.

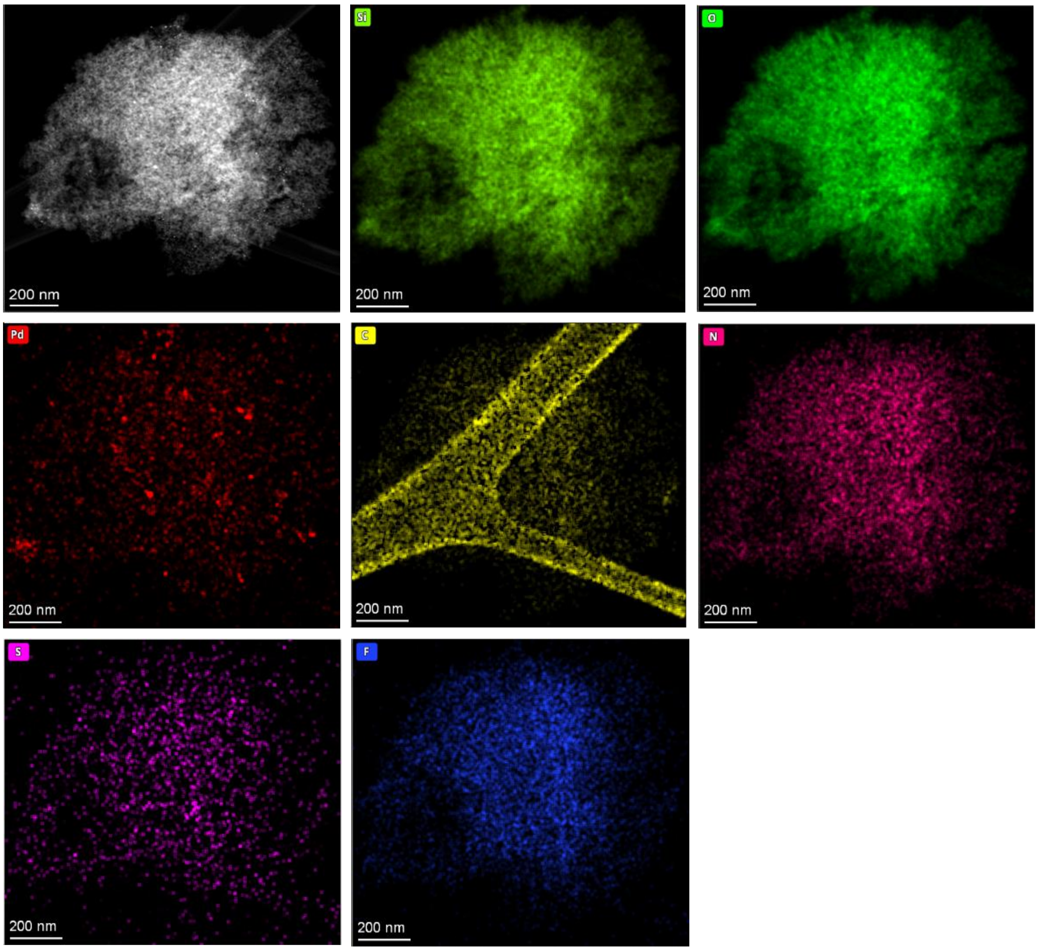

Figure S25-1. TEM-EDX mapping images of 40w.-% $[NTf_2]^-$-SCILL revealing homogeneous distribution of the ionic liquid throughout the whole support.

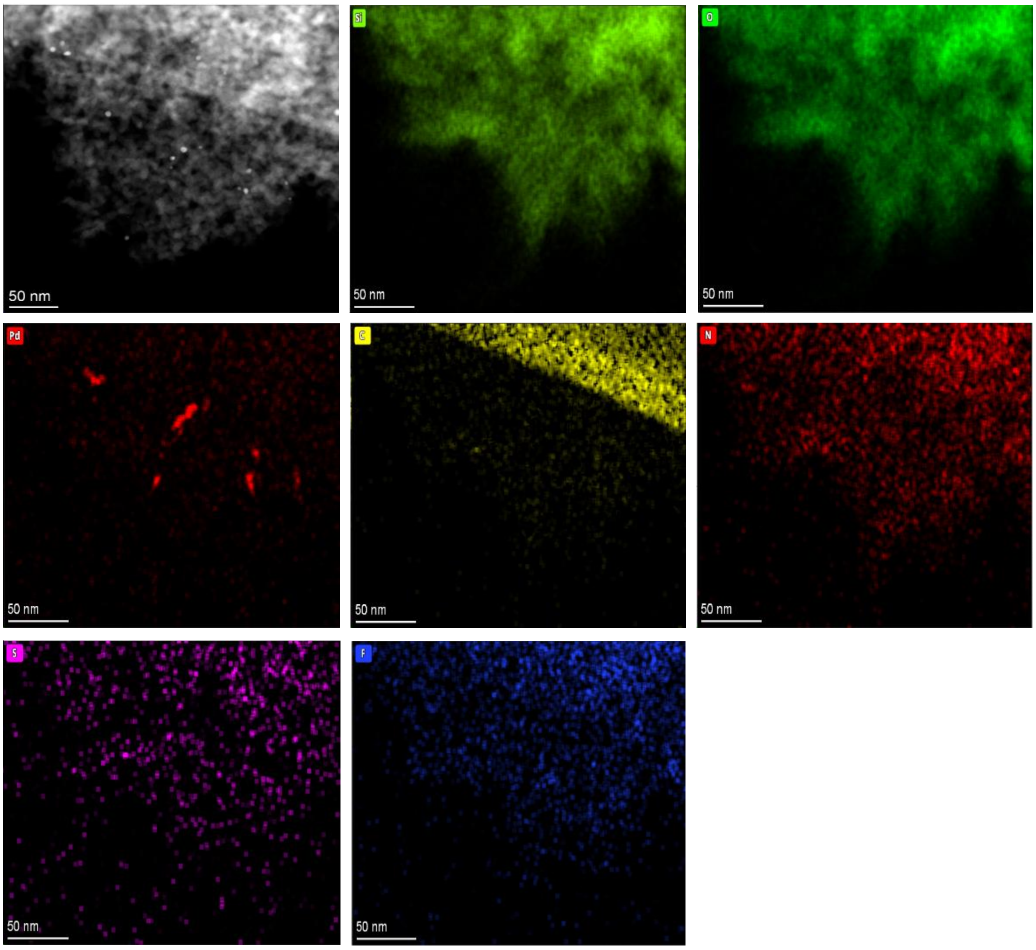

Figure S25-2. TEM-EDX mapping images of 40w.-% $[NTf_2]^-$-SCILL revealing homogeneous distribution of the ionic liquid throughout the whole support.

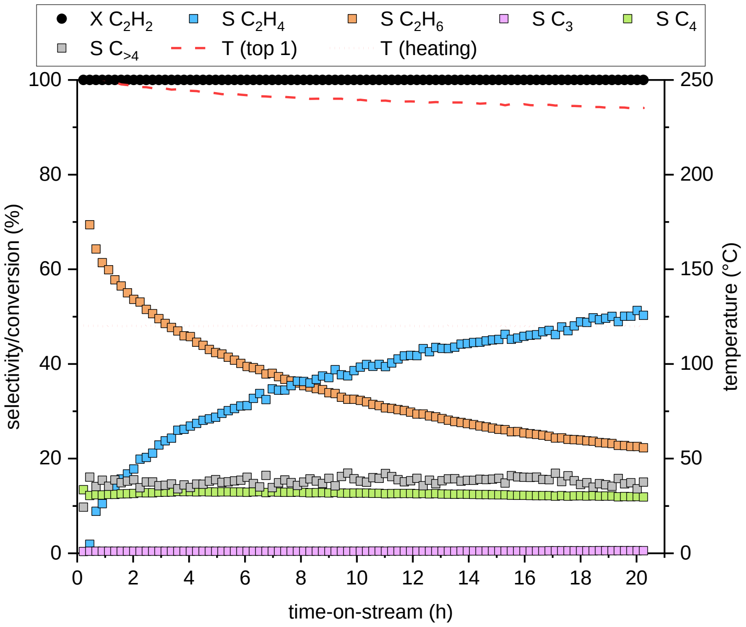

Figure S26. Ethylene, ethane, C_3, C_4 , $C_{>4}$ selectivity and acetylene conversion of 40 w.-% $[NTf_2]^-$-SCILL (Figure 3b) as well as temperature of heating and at different positions in the catalyst bed (top 1, top 2, middle, bottom) versus time-on-stream in the selective hydrogenation of concentrated acetylene streams ($C_2H_2/C_2H_4/H_2$ 1:1:5, 10 bar, 120 °C, X_{C2H2} = 100%, CB ≥ 0.97, WHSV 42 000 $cm^3\ h^{-1}\ g^{-1}_{cat}$).

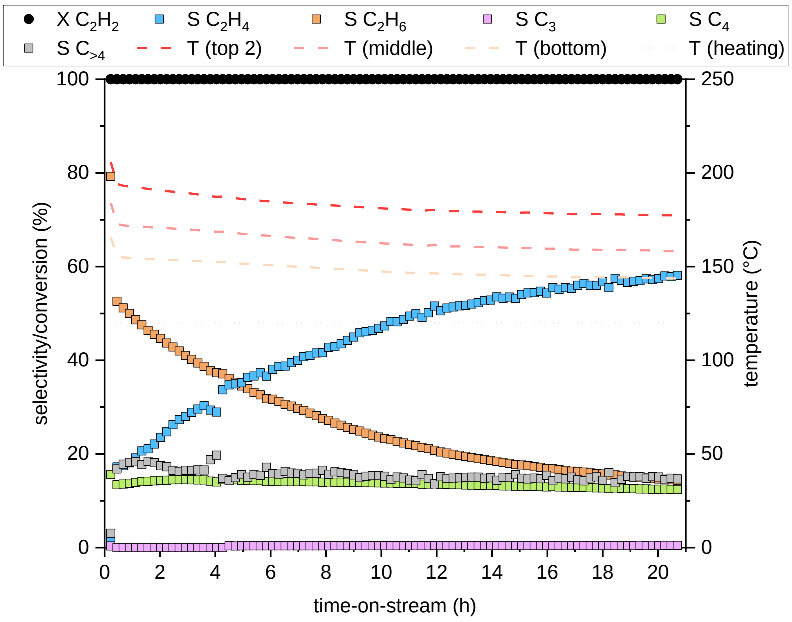

Figure S27. Ethylene, ethane, C_3, C_4 , $C_{>4}$ selectivity and acetylene conversion of 80 w.-% $[NTf_2]^-$-SCILL (Figure 3b) as well as temperature of heating and at different positions in the catalyst bed (top 1, top 2, middle, bottom) versus time-on-stream in the selective hydrogenation of concentrated acetylene streams ($C_2H_2/C_2H_4/H_2$ 1:1:5, 10 bar, 120 °C, X_{C2H2} = 100%, CB ≥ 0.97, WHSV 42 000 $cm^3\ h^{-1}\ g^{-1}_{cat}$).

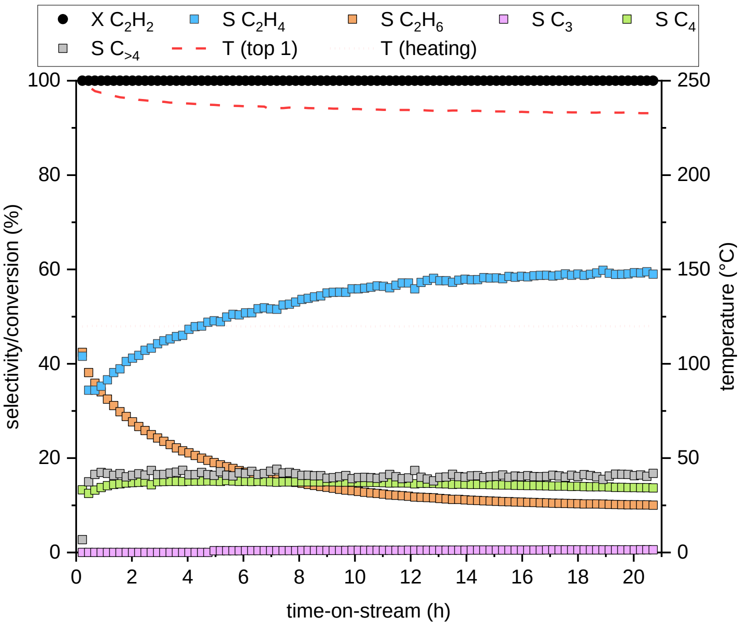

Figure S28. Ethylene, ethane, C_3, C_4 , $C_{>4}$ selectivity and acetylene conversion of 120 w.-% $[NTf_2]^-$-SCILL (Figure 3b) as well as temperature of heating and at different positions in the catalyst bed (top 1, top 2, middle, bottom) versus time-on-stream in the selective hydrogenation of concentrated acetylene streams ($C_2H_2/C_2H_4/H_2$ 1:1:5, 10 bar, 120 °C, X_{C2H2} = 100%, CB ≥ 0.97, WHSV 42 000 $cm^3\ h^{-1}\ g^{-1}_{cat}$).

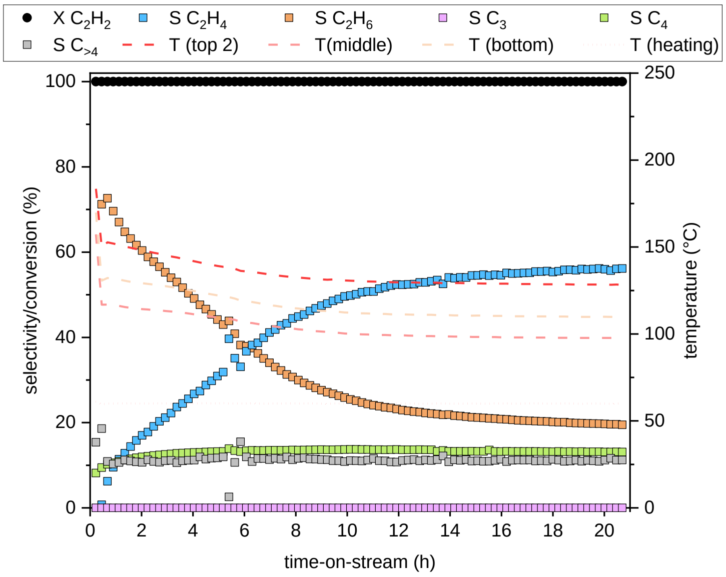

Figure S29. Ethylene, ethane, C_3, C_4 , $C_{>4}$ selectivity and acetylene conversion of $[NTf_2]^-$-SCILL (Figure 3c) as well as temperature of heating and at different positions in the catalyst bed (top 1, top 2, middle, bottom) versus time-on-stream in the selective hydrogenation of concentrated acetylene streams ($C_2H_2/C_2H_4/H_2$ 1:1:5, 10 bar, 60 °C, X_{C2H2} = 100%, CB ≥ 0.97, WHSV 42 000 cm^3 h^{-1} g^{-1}_{cat}).

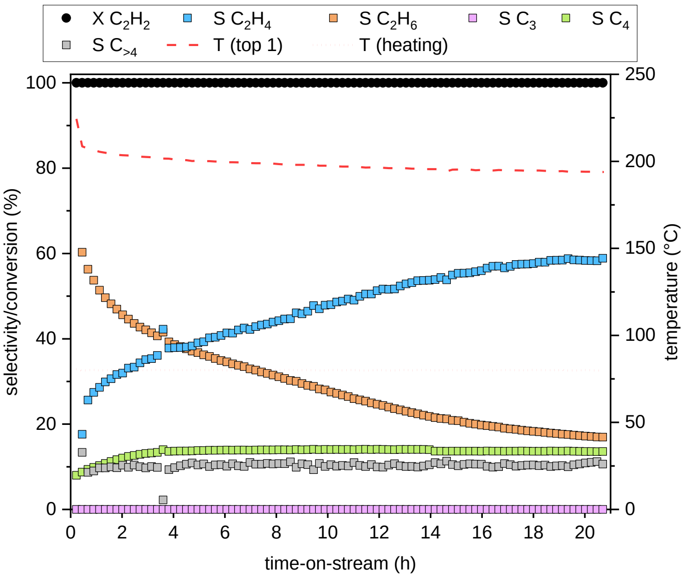

Figure S30. Ethylene, ethane, C_3, C_4 , $C_{>4}$ selectivity and acetylene conversion of $[NTf_2]^-$-SCILL (Figure 3c) as well as temperature of heating and at different positions in the catalyst bed (top 1, top 2, middle, bottom) versus time-on-stream in the selective hydrogenation of concentrated acetylene streams ($C_2H_2/C_2H_4/H_2$ 1:1:5, 10 bar, 80 °C, X_{C2H2} = 100%, CB ≥ 0.97, WHSV 42 000 cm^3 h^{-1} g^{-1}_{cat}).

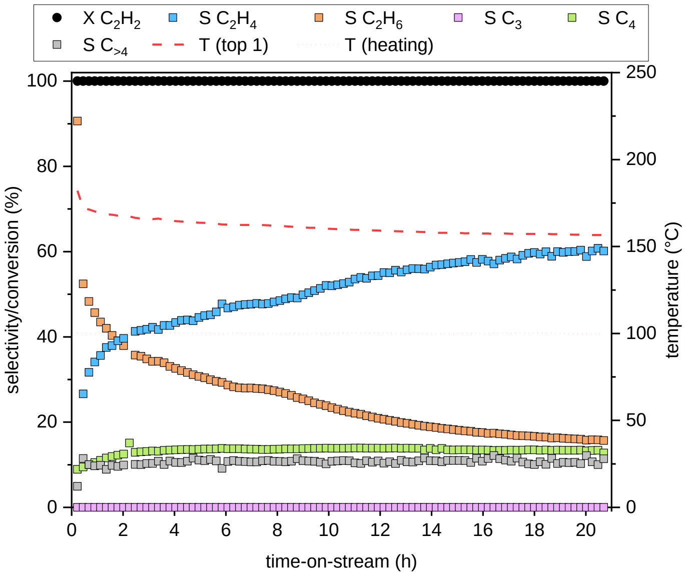

Figure S31. Ethylene, ethane, C_3, C_4 , $C_{>4}$ selectivity and acetylene conversion of $[NTf_2]^-$-SCILL (Figure 3c) as well as temperature of heating and at different positions in the catalyst bed (top 1, top 2, middle, bottom) versus time-on-stream in the selective hydrogenation of concentrated acetylene streams ($C_2H_2/C_2H_4/H_2$ 1:1:5, 10 bar, 100 °C, X_{C2H2} = 100%, CB ≥ 0.97, WHSV 42 000 cm^3 h^{-1} g^{-1}_{cat}).

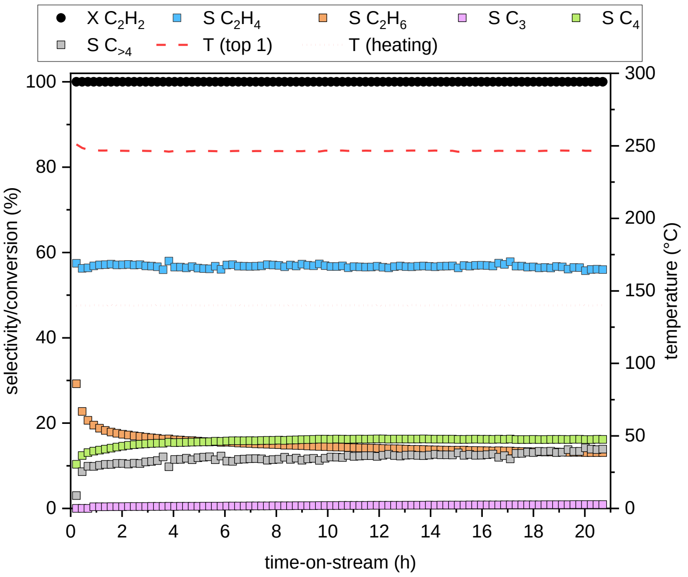

Figure S32. Ethylene, ethane, C_3, C_4 , $C_{>4}$ selectivity and acetylene conversion of $[NTf_2]^-$-SCILL (Figure 3c) as well as temperature of heating and at different positions in the catalyst bed (top 1, top 2, middle, bottom) versus time-on-stream in the selective hydrogenation of concentrated acetylene streams ($C_2H_2/C_2H_4/H_2$ 1:1:5, 10 bar, 140 °C, X_{C2H2} = 100%, CB ≥ 0.97, WHSV 42 000 cm^3 h^{-1} g^{-1}_{cat}).

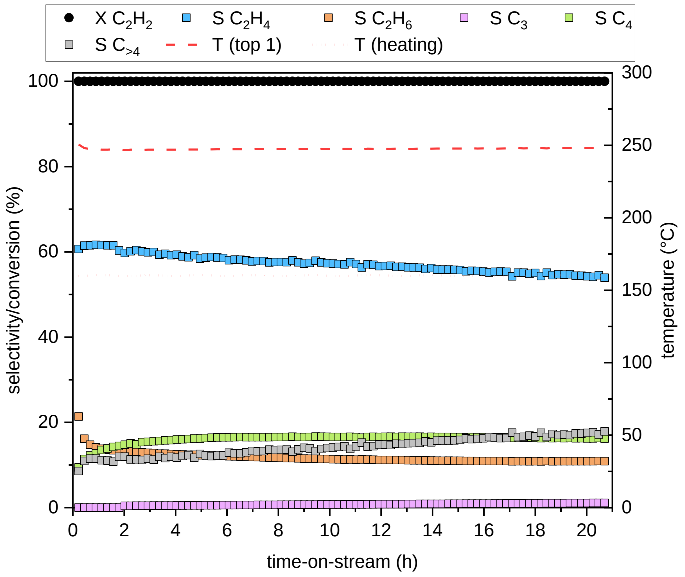

Figure S33. Ethylene, ethane, C_3, C_4 , $C_{>4}$ selectivity and acetylene conversion of $[NTf_2]^-$-SCILL (Figure 3c) as well as temperature of heating and at different positions in the catalyst bed (top 1, top 2, middle, bottom) versus time-on-stream in the selective hydrogenation of concentrated acetylene streams ($C_2H_2/C_2H_4/H_2$ 1:1:5, 10 bar, 160 °C, X_{C2H2} = 100%, CB ≥ 0.97, WHSV 42 000 cm^3 h^{-1} g^{-1}_{cat}).

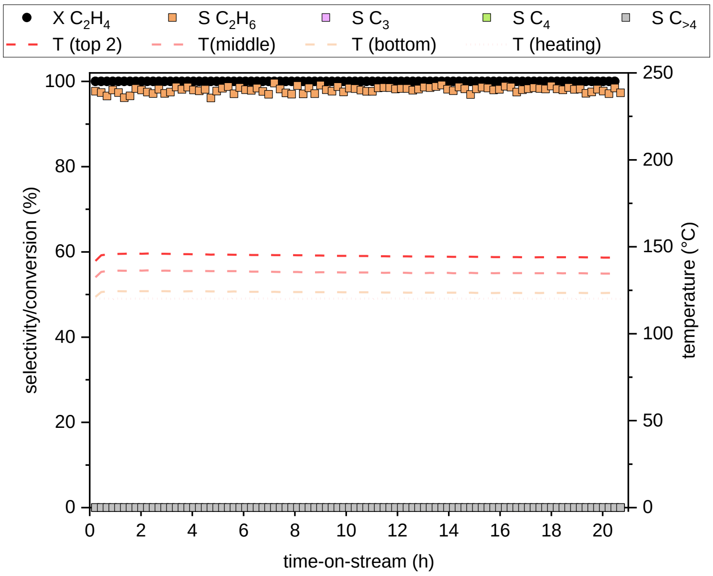

Figure S34. Ethane, C_3, C_4 , $C_{>4}$ selectivity and ethylene conversion of SC (Figure 4a) as well as temperature of heating and at different positions in the catalyst bed (top 1, top 2, middle, bottom) versus time-on-stream in the selective hydrogenation of concentrated ethylene streams ($N_2/C_2H_4/H_2$ 1:1:5, 10 bar, 120 °C, X_{C2H2} = 100%, CB ≥ 0.98, WHSV 42 000 cm^3 h^{-1} g^{-1}_{cat}).

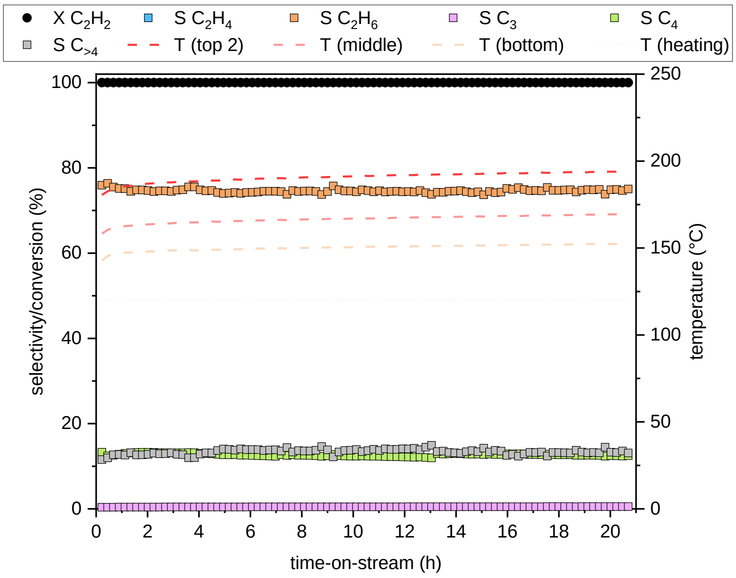

Figure S35. Ethylene, ethane, C_3, C_4 , $C_{>4}$ selectivity and acetylene conversion of SC (Figure 4a) as well as temperature of heating and at different positions in the catalyst bed (top 1, top 2, middle, bottom) versus time-on-stream in the selective hydrogenation of concentrated acetylene streams ($C_2H_2/N_2/H_2$ 1:1:5, 10 bar, 120 °C, X_{C2H2} = 100%, CB ≥ 0.87, WHSV 42 000 cm^3 h^{-1} g^{-1}_{cat}).

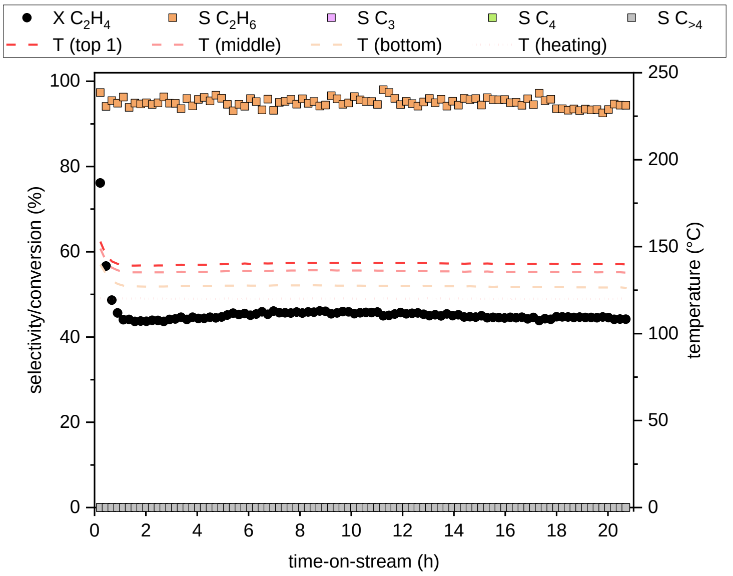

Figure S36. Ethane, C_3, C_4 , $C_{>4}$ selectivity and ethylene conversion of $[NTf_2]^-$-SCILL (Figure 4a) as well as temperature of heating and at different positions in the catalyst bed (top 1, top 2, middle, bottom) versus time-on-stream in the selective hydrogenation of concentrated ethylene streams ($N_2/C_2H_4/H_2$ 1:1:5, 10 bar, 120 °C, X_{C2H2} = 100%, CB ≥ 0.98, WHSV 42 000 cm^3 h^{-1} g^{-1}_{cat}).

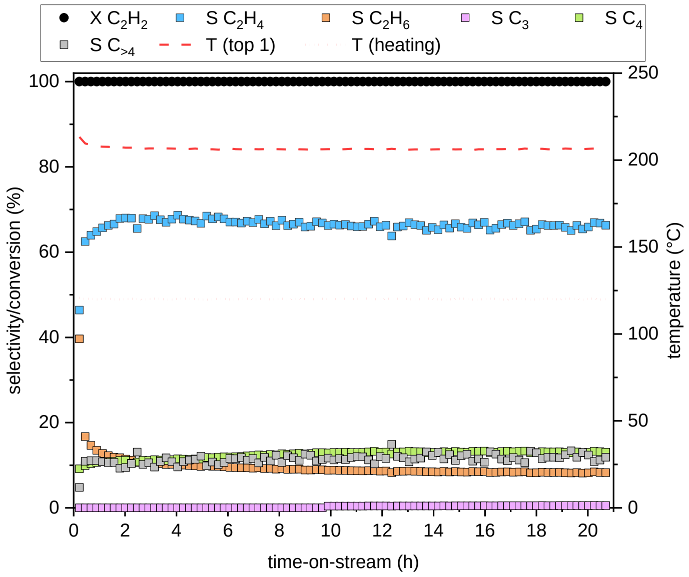

Figure S37. Ethylene, ethane, C_3, C_4 , $C_{>4}$ selectivity and acetylene conversion of $[NTf_2]^-$-SCILL (Figure 4a) as well as temperature of heating and at different positions in the catalyst bed (top 1, top 2, middle, bottom) versus time-on-stream in the selective hydrogenation of concentrated acetylene streams ($C_2H_2/N_2/H_2$ 1:1:5, 10 bar, 120 °C, X_{C2H2} = 100%, CB ≥ 0.89, WHSV 42 000 $cm^3\ h^{-1}\ g^{-1}_{cat}$).

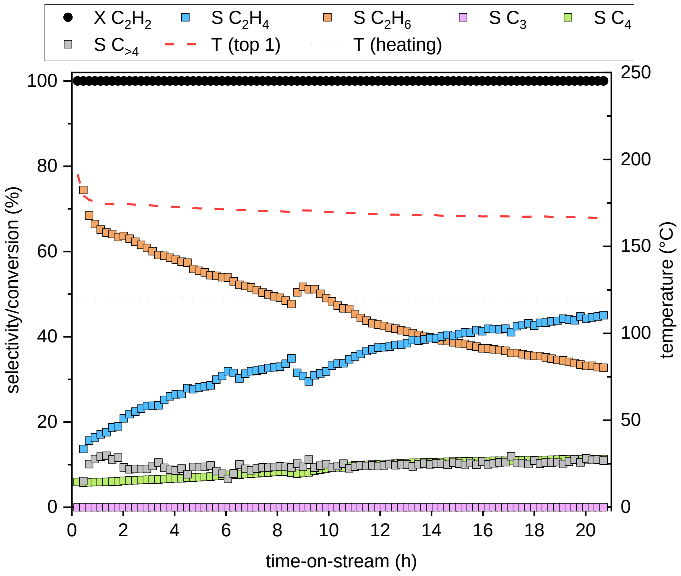

Figure S38. Ethylene, ethane, C_3, C_4 , $C_{>4}$ selectivity and acetylene conversion of $[NTf_2]^-$-SCILL (Figure 4b) as well as temperature of heating and at different positions in the catalyst bed (top 1, top 2, middle, bottom) versus time-on-stream in the selective hydrogenation of concentrated acetylene streams ($C_2H_2/C_2H_2/H_2/$ N_2 1:1:10:0, 10 bar, 120 °C, X_{C2H2} = 100%, CB ≥ 0.95, WHSV 36 000 $cm^3\ h^{-1}\ g^{-1}_{cat}$).

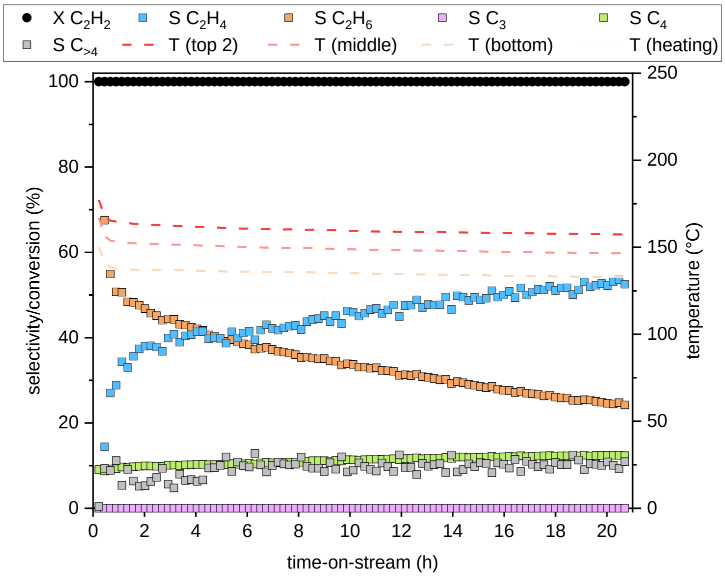

Figure S39. Ethylene, ethane, C_3, C_4 , $C_{>4}$ selectivity and acetylene conversion of [NTf$_2$]$^-$-SCILL (Figure 4b) as well as temperature of heating and at different positions in the catalyst bed (top 1, top 2, middle, bottom) versus time-on-stream in the selective hydrogenation of concentrated acetylene streams (C_2H_2/C_2H_2/H_2/ N_2 1:1:7:3, 10 bar, 120 °C, X_{C2H2} = 100%, CB ≥ 0.95, WHSV 36 000 cm^3 h^{-1} g$^{-1}_{cat}$).

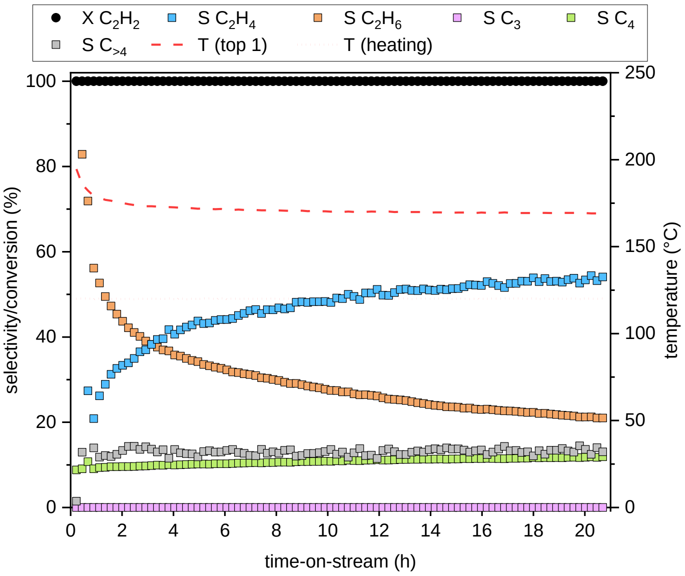

Figure S40. Ethylene, ethane, C_3, C_4 , $C_{>4}$ selectivity and acetylene conversion of [NTf$_2$]$^-$-SCILL (Figure 4b) as well as temperature of heating and at different positions in the catalyst bed (top 1, top 2, middle, bottom) versus time-on-stream in the selective hydrogenation of concentrated acetylene streams (C_2H_2/C_2H_2/H_2/ N_2 1:1:5:5, 10 bar, 120 °C, X_{C2H2} = 100%, CB ≥ 0.94, WHSV 36 000 cm^3 h^{-1} g$^{-1}_{cat}$).

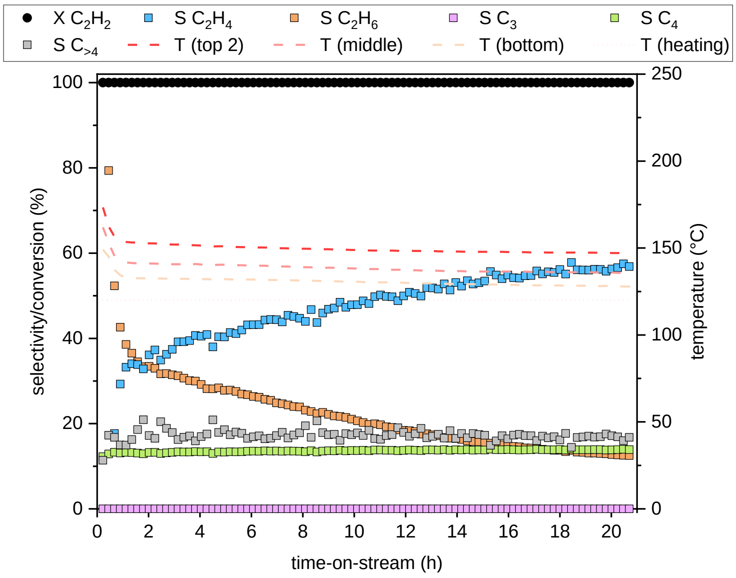

Figure S41. Ethylene, ethane, C_3, C_4 , $C_{>4}$ selectivity and acetylene conversion of $[NTf_2]^-$-SCILL (Figure 4b) as well as temperature of heating and at different positions in the catalyst bed (top 1, top 2, middle, bottom) versus time-on-stream in the selective hydrogenation of concentrated acetylene streams ($C_2H_2/C_2H_2/H_2/$ N_2 1:1:3:7, 10 bar, 120 °C, X_{C2H2} = 100%, CB ≥ 0.91, WHSV 36 000 cm^3 h^{-1} g^{-1}_{cat}).

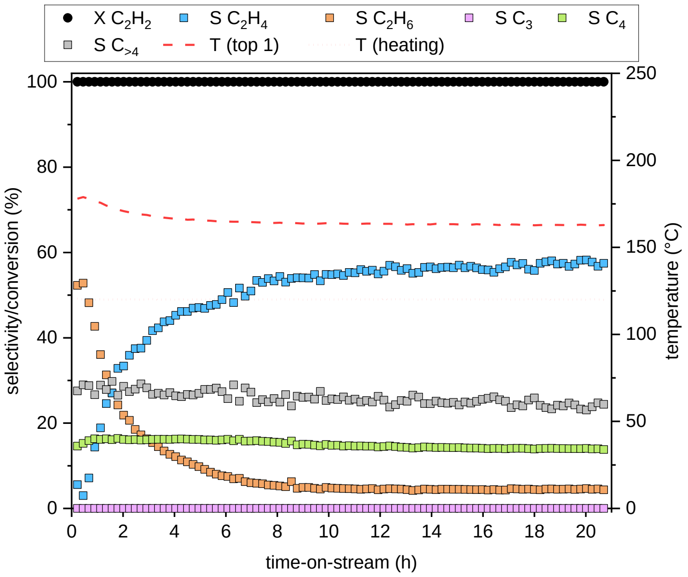

Figure S42. Ethylene, ethane, C_3, C_4 , $C_{>4}$ selectivity and acetylene conversion of $[NTf_2]^-$-SCILL (Figure 4b) as well as temperature of heating and at different positions in the catalyst bed (top 1, top 2, middle, bottom) versus time-on-stream in the selective hydrogenation of concentrated acetylene streams ($C_2H_2/C_2H_2/H_2/$ N_2 1:1:1.5:8.5, 10 bar, 120 °C, X_{C2H2} = 100%, CB ≥ 0.87, WHSV 36 000 cm^3 h^{-1} g^{-1}_{cat}).

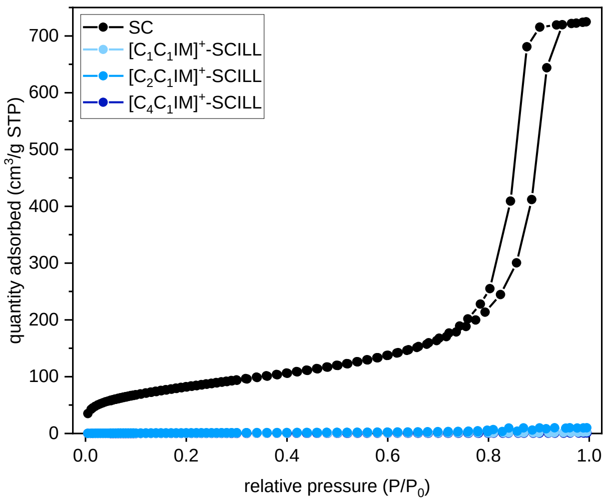

Figure S43. N_2 physisorption isotherms of SC and $[C_xC_1IM][Me_2PO_4]$-SCILLs showing the complete pore filling of the silica gel support.

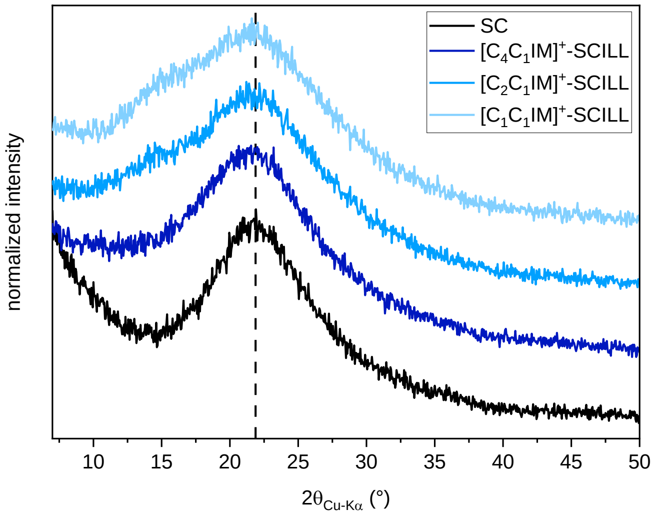

Figure S44. XRD pattern of SC and $[C_xC_1IM][Me_2PO_4]$-SCILLs revealing reflections resulting from the ordering of cations and anions in the ionic liquid phase.

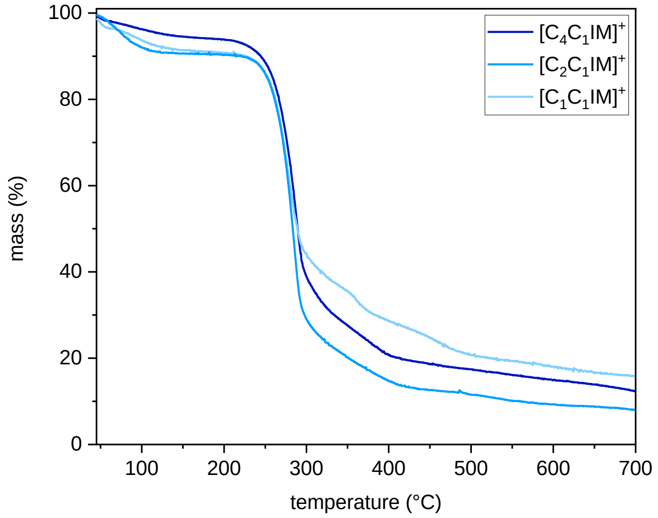

Figure S45. TGA decomposition curves of [C_xC_1IM][Me_2PO_4] at 5 °C/min heating rate and 40 mL/min air.

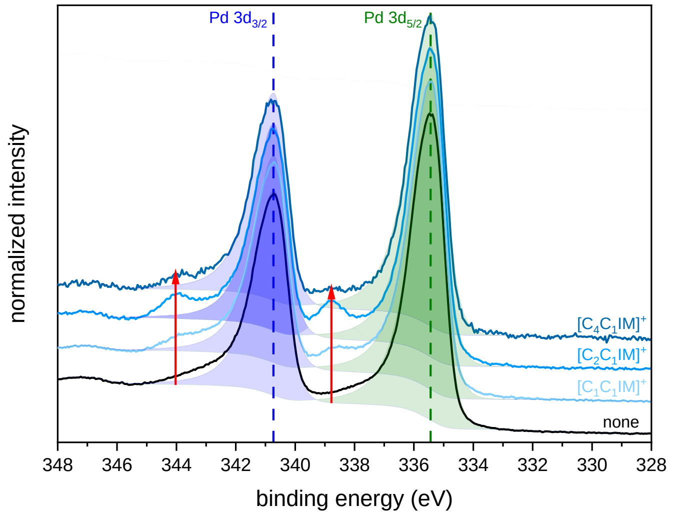

Figure S46. XPS spectra of Pd 3d region of Pd model catalysts surface coated with [C_xC_1IM][Me_2PO_4]. Red arrow indicate the upcoming of an oxidized Pd species at higher binding energies depending on the alkyl chain length in the cation.

Variation of Cations

Since the hydrogen (and ethylene) gas solubility in ionic liquids is influenced by the available free volume between anions and cations[3, 7, 16], it should be possible to improve the selectivity performance of a SCILL with a specific anion by modifying the cation accordingly. For this reason, the effect of decreasing alkyl chain length of the cation (C_4 > C_2 > C_1) and thereby adapting it to the size of the anion was studied for the well-performing [Me_2PO_4]$^-$-SCILL. Details on characterization can be found here in the supporting information (Tables S8-11, Figure S43-46). Even though the observed effect is only slightly above the error bars, ethylene selectivity stepwise improves, as expected, from 76 to 79% via reduced full hydrogenation to ethane from 14 to 9% while the formation of oligomers increases from 10 to 12% when the alkyl chain length on the cation is shortened (Figures S47, S10, S48-49 and Table S12). The cation can thus be used to fine-tune the performance of a SCILL.

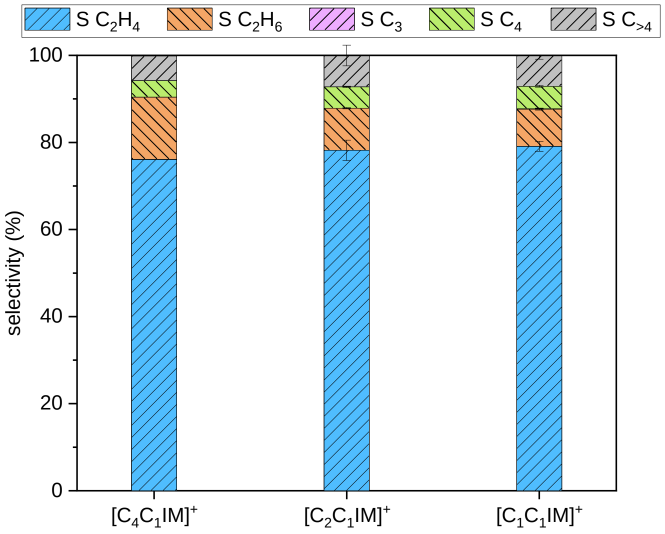

Figure S47. Average ethylene, ethane, C_3, C_4 and $C_{>4}$ selectivity of $[Me_2PO_4]^-$-SCILL (0-2 h on-stream, $C_2H_2/C_2H_4/H_2$ 1:1:5, 10 bar, 120 °C, X_{C2H2} = 100%, CB ≥ 0.95, WHSV 42 000 cm^3 h^{-1} g^{-1}_{cat}) under variation of alkyl chain length on cation.

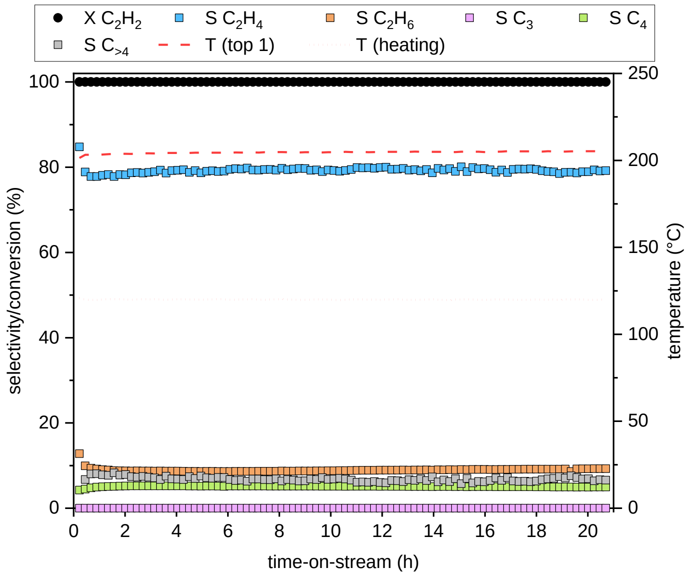

Figure S48. Ethylene, ethane, C_3, C_4 , $C_{>4}$ selectivity and acetylene conversion of $[C_2C_1IM][Me_2PO_4]$-SCILL (Figure S47) as well as temperature of heating and at different positions in the catalyst bed (top 1, top 2, middle, bottom) versus time-on-stream in the selective hydrogenation of concentrated acetylene streams ($C_2H_2/C_2H_4/H_2$ 1:1:5, 10 bar, 120 °C, X_{C2H2} = 100%, CB ≥ 0.97, WHSV 42 000 cm^3 h^{-1} g^{-1}_{cat}).

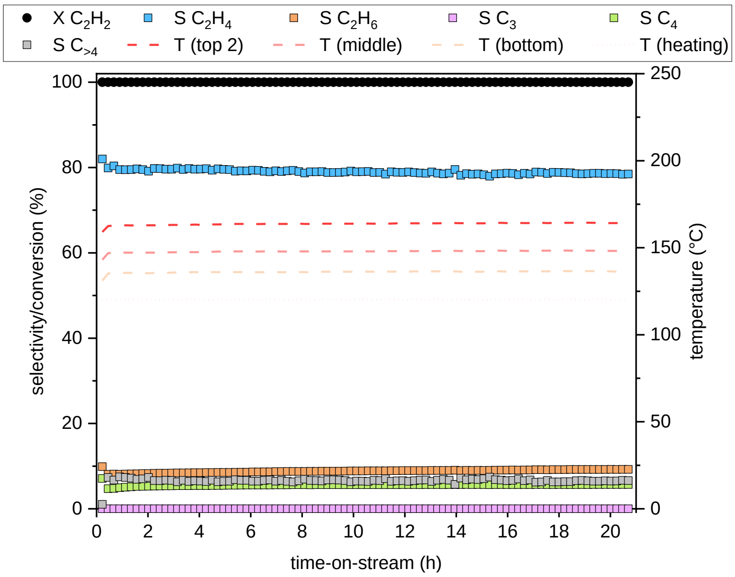

Figure S49. Ethylene, ethane, C_3, C_4 , $C_{>4}$ selectivity and acetylene conversion of [C_1C_1IM][Me_2PO_4]-SCILL (Figure S47) as well as temperature of heating and at different positions in the catalyst bed (top 1, top 2, middle, bottom) versus time-on-stream in the selective hydrogenation of concentrated acetylene streams ($C_2H_2/C_2H_4/H_2$ 1:1:5, 10 bar, 120 °C, X_{C2H2} = 100%, CB ≥ 0.97, WHSV 42 000 cm^3 h^{-1} g^{-1}_{cat}).

Literature

[1] I.-T. Trotus, PhD thesis, Ruhr-Universität Bochum **2016**.

[2] a) K. S. Kley, J. De Bellis, F. Schüth, *Catal. Sci. Technol.* **2022**, 1-13; b) Ö. T. Agbaba, Ioan-Teodor; Schmidt, Wolfgang; Schüth, Ferdi, *Ind Eng Chem Res* **2023**, *62*, 1819-1825; c) I. T. Trotus, T. Zimmermann, N. Duyckaerts, J. Geboers, F. Schuth, *Chem Commun* **2015**, *51*, 7124-7127.

[3] J. Palgunadi, H. S. Kim, J. M. Lee, S. Jung, *Chem Eng Process* **2010**, *49*, 192-198.

[4] J. Kumelan, A. P. S. Kamps, D. Tuma, G. Maurer, *Fluid Phase Equilibr* **2007**, *260*, 3-8.

[5] S. Jung, J. Palgunadi, J. H. Kim, H. Lee, B. S. Ahn, M. Cheong, H. S. Kim, *J Membrane Sci* **2010**, *354*, 63-67.

[6] M. Ruta, G. Laurenczy, P. J. Dyson, L. Kiwi-Minsker, *J Phys Chem C* **2008**, *112*, 17814-17819.

[7] J. Palgunadi, S. Y. Hong, J. K. Lee, H. Lee, S. D. Lee, M. Cheong, H. S. Kim, *J Phys Chem B* **2011**, *115*, 1067-1074.

[8] P. J. Dyson, G. Laurenczy, C. A. Ohlin, J. Vallance, T. Welton, *Chem Commun* **2003**, 2418-2419.

[9] Q. T. Wang, Y. Q. Xu, J. Zhou, L. Y. Xu, L. Yu, D. H. Jiang, C. S. Lu, Z. Y. Pan, Q. F. Zhang, X. N. Li, *J Ind Eng Chem* **2021**, *93*, 448-460.

[10] Iolitec Ionic Liquids Technologies GmbH, "Catalogue Products", can be found under https://iolitec.de/index.php/products/ionic_liquids/catalogue, **2024** (accessed 23.04.2024).

[11] A. F. M. Cláudio, L. Swift, J. P. Hallett, T. Welton, J. A. P. Coutinho, M. G. Freire, *Phys Chem Chem Phys* **2014**, *16*, 6593-6601.

[12] Y. Marcus, *J Mol Liq* **2015**, *209*, 289-293.

[13] H. D. B. Jenkins, J. F. Liebman, *Inorg Chem* **2005**, *44*, 6359-6372.

[14] H. P. Steinruck, P. Wasserscheid, *Catal Lett* **2015**, *145*, 380-397.

[15] A. Kadyan, S. Pandey, *J Phys Chem B* **2017**, *121*, 1081-1091.

[16] Z. G. Lei, C. N. Dai, B. H. Chen, *Chem. Rev.* **2014**, *114*, 1289-1326.
